# CXCL6 exacerbates metabolic dysfunction-associated steatohepatitis by suppressing LPIN1-mediated fatty acid oxidation in hepatocytes

**DOI:** 10.7150/ijbs.129358

**Published:** 2026-05-01

**Authors:** Ye Eun Cho, Min-Ju Kim, Yeonsoo Kim, Hyeokjin Lim, Yunseo Park, Sangok Kim, Seung-Jin Kim, Jin-Wook Yoo, Seungjin Ryu, Parkyong Song, Changwan Hong, Yong He, Haeseung Lee, Je-Yoel Cho, Seonghwan Hwang

**Affiliations:** 1 College of Pharmacy and Research Institute for Drug Development, Pusan National University, Busan 46241, Republic of Korea.; 2 Korea Bioinformation Center, Korea Research Institute of Bioscience & Biotechnology, Daejeon 34141, Republic of Korea.; 3 Department of Biochemistry, College of Natural Sciences, Kangwon National University, Chuncheon 24341, Republic of Korea.; 4 Department of Biochemistry, Chung-Ang University College of Medicine, Seoul 06974, Republic of Korea.; 5 Department of Convergence Medicine, Pusan National University School of Medicine, Yangsan 50612, Republic of Korea.; 6 Department of Anatomy, Pusan National University School of Medicine, Yangsan 50612, Republic of Korea; 7 Department of Convergence Medical Science, Pusan National University School of Medicine, Yangsan 50612, Republic of Korea.; 8 State Key Laboratory of Drug Research, Shanghai Institute of Materia Medica (SIMM), Chinese Academy of Sciences, Shanghai 201203, China.; 9 Department of Biochemistry, Brain Korea 21 Project and Research Institute for Veterinary Science, Seoul National University, College of Veterinary Medicine, Seoul 08826, Republic of Korea.; 10 Comparative Medicine Disease Research Center (CDRC), Science Research Center (SRC), Seoul National University, Seoul 08826, Republic of Korea.

**Keywords:** chemokine, fatty acid oxidation, lipotoxicity, steatosis, hepatocyte

## Abstract

Progression of hepatic steatosis to metabolic dysfunction-associated steatohepatitis (MASH) is driven by impaired fatty acid (FA) oxidation and subsequent hepatocyte lipotoxicity. While MASH is characterized by upregulation of the neutrophil chemoattractant CXCL6, which functions through CXCR2, the direct impact of this pathway on hepatocyte FA metabolism during MASH progression remains unclear. Here, we demonstrate that hepatic overexpression of *Cxcl5* (the murine homolog of *CXCL6*) inhibits hepatocyte FA oxidation and promotes MASH progression in mice. In contrast, *Cxcl5* deficiency conferred protection against diet-induced MASH by reducing hepatic FA levels and restoring the expression of LPIN1, a transcriptional coactivator of PPARα, thereby normalizing FA metabolic gene expression. Mechanistically, CXCL6 activated JNK, leading to the inhibitory phosphorylation of the glucocorticoid receptor (GR). This blockade prevented GR-dependent activation of the LPIN1 promoter, thereby suppressing the LPIN1-PPARα axis in hepatocytes. *Lpin1* knockdown reversed the protective phenotype in *Cxcl5*-deficient mice, confirming that LPIN1 suppression is the essential driver of CXCL6-mediated MASH progression. Consistently, human MASH samples exhibited reduced LPIN1 expression, which inversely correlated with CXCL6 expression. In conclusion, beyond its canonical role in neutrophil recruitment, CXCL6 promotes MASH progression by inhibiting the GR-LPIN1-PPARα axis in hepatocytes, resulting in impaired FA oxidation and lipotoxicity.

## Introduction

Metabolic dysfunction-associated steatotic liver disease (MASLD) encompasses a continuum of liver disorders driven by metabolic abnormalities. This ranges from simple steatosis to metabolic dysfunction-associated steatohepatitis (MASH), cirrhosis, and hepatocellular carcinoma. Globally, MASLD is estimated to affect approximately 25% of the adult population, with MASH affecting approximately 5-6% of the population. MASH occurrences continue to increase and are becoming a leading contributor to end-stage liver disease.

Steatosis is the initial manifestation of MASLD and generally considered benign, because it is not accompanied by substantial hepatic injury or inflammation. Approximately 25% of individuals with steatosis develop MASH, a more severe form with hepatocellular injury, inflammation, and fibrosis. The liver uses several metabolic strategies to limit the toxic accumulation of free fatty acids (FFAs), including mitochondrial β-oxidation that catabolizes fatty acids (FAs) into acetyl-CoA. However, under pathological conditions that favor MASLD progression, these protective mechanisms become insufficient, resulting in elevated intracellular FFA levels. The consequent lipotoxic stress contributes to hepatocellular dysfunction and transition from steatosis to MASH. Mitochondrial function is compromised, and FA oxidation is disrupted in experimental models and clinical cases of MASH 1. The expression of genes involved in hepatic β-oxidation is primarily influenced by the transcriptional activity of peroxisome proliferator-activated receptor α (PPARα), whose expression is reduced in patients with MASH 2.

Lipin-1 (LPIN1) is a phosphatidic acid phosphatase (PAP) that converts phosphatidic acid into diacylglycerol 3. In addition to its enzymatic role, LPIN1 functions as a transcriptional coregulator that modulates FA metabolism. LPIN1 interacts with transcription factors such as PPARs to regulate their activity and downstream gene expression. Finck et al. demonstrated that LPIN1 enhances PPARα activity by upregulating its transcription and functioning as a transcriptional coactivator with PPARγ coactivator 1α (PGC1α) 4. PGC1α is a key coactivator that facilitates PPARα-driven transcription of genes associated with mitochondrial FA β-oxidation 5. In addition, LPIN1 has been implicated in the suppression of lipogenesis. Peterson et al. reported that LPIN1 suppresses FA synthesis by modulating the mechanistic target of rapamycin complex 1 (mTORC1) signaling and downregulating sterol regulatory element-binding proteins (SREBPs) 6. These reports support the critical role of LPIN1 as a regulator of FA metabolism. However, the role of the LPIN1-PPARα pathway in the progression of steatosis to MASH remains unclear.

The progression of steatosis to MASH is associated with increased hepatic neutrophil infiltration, which is not typically observed in simple steatosis in obese individuals or mice fed a high-fat diet (HFD) 7, 8. Neutrophil-recruiting chemokines, such as CXC motif chemokine ligand (CXCL)1 and CXCL8, are upregulated during the transition to MASH. Our previous studies demonstrated that hepatic overexpression of CXCL1 or CXCL8 augments hepatic neutrophil infiltration and accelerates steatosis-to-MASH progression in HFD-fed mice 9, 10. In addition to CXCL1 and CXCL8, several other chemokines promote neutrophil infiltration, such as CXCL2 and CXCL6.

Analysis of multiple RNA sequencing (RNA-seq) datasets in the current study demonstrated that CXCL1, CXCL6, and CXCL8 are neutrophil-recruiting chemokines upregulated during the transition from steatosis to MASH in humans. The expression of hepatic CXCL6 was higher than that of CXCL1 and CXCL8, which are well-established neutrophil-recruiting chemokines. However, despite its relatively high expression, the involvement of CXCL6 in the steatosis-to-MASH transition has been considerably less explored than the involvement of CXCL1 and CXCL8.

CXCL6 is an ELR^+^ CXC chemokine that recruits neutrophils by binding to CXCR1 and CXCR2, both of which are expressed on neutrophils 11-13. The mouse genome lacks an annotated *Cxcl6* gene. Instead, *Cxcl5* is the functional murine homolog of human *CXCL6*. Consistent with this relationship, mouse CXCL5 has a greater amino acid sequence similarity to human CXCL6 than to human CXCL5 14.

Despite the potential relevance of CXCL6, its *in vivo* contribution to MASH development has not yet been investigated using genetic ablation or overexpression of *Cxcl5* in mice. Here, we address this knowledge gap using gain- and loss-of-function approaches targeting *Cxcl5*. Adenovirus-mediated hepatic *Cxcl5* overexpression accelerated the progression of steatosis to MASH in mice, whereas *Cxcl5* deficiency protected against MASH progression. CXCL6 suppressed LPIN1-mediated PPARα activation and impaired FA oxidation in hepatocytes. This indicates that CXCL6, beyond its canonical role in neutrophil recruitment, promotes MASH progression by directly exacerbating hepatocyte lipotoxicity.

## Materials & Methods

### Collection of transcriptomic datasets from liver tissues of patients with MASLD

Publicly available transcriptomic datasets from liver tissues of patients with MASLD were obtained from the Gene Expression Omnibus (GEO) database. Bulk RNA-seq datasets (GSE167523 and GSE135251) included read counts and transcripts per million (TPM) values. Single-cell RNA-seq datasets (GSE136103 and GSE244832) included read count matrices.

### Bulk RNA sequencing

Wild-type and *Cxcl5*-deficient mice were fed a gubra amylin (AMLN) diet (40% kcal derived from fat, supplemented with fructose and cholesterol; #D09100310, Research Diets, New Brunswick, NJ, USA) for 20 weeks, and total RNA was extracted from whole liver tissues using RiboEX reagent (#301-002; Geneall, Seoul, Republic of Korea) according to the manufacturer's instructions. RNA sequencing was performed using the Illumina Novaseq 6000 platform. Raw sequencing reads were first assessed for quality using FastQC (v0.11.9). Adapter trimming and quality filtering were conducted using Trim Galore (v0.6.6). Filtered reads were aligned to the Mus musculus reference genome (GRCm39, Ensembl release 104) using STAR (v2.7.9a). Gene-level read counts were generated using RSEM (v1.3.3). Bulk RNA-seq data have been deposited in the NCBI GEO under accession number GSE325102.

### Differential expression analysis with bulk RNA-seq data

Bulk RNA-seq read count data were processed using the DESeq2 R package (v1.48.1) to identify genes exhibiting stage-dependent expression patterns across disease progression. Pairwise comparisons between two groups were conducted using the Wald test. For analyses involving multiple disease stages, a likelihood ratio test was applied by modeling disease stage as a continuous variable, enabling identification of genes with consistent directional changes relative to healthy controls. Resulting *p-*values were corrected for multiple comparisons using the Benjamini-Hochberg procedure.

Chemokine genes (defined as those with CXCL or CCL prefixes) were filtered according to the following criteria: (i) BH-adjusted *p*-values below 0.01 in both datasets, and (ii) log_2_ fold change greater than 1. Expression differences of the selected chemokines across disease groups were evaluated using the Kruskal-Wallis test, followed by pairwise Wilcoxon rank-sum tests for post hoc analysis.

### Functional enrichment analysis

Biological function enrichment was assessed using both over-representation analysis (ORA) and gene set enrichment analysis (GSEA) based on Gene Ontology (GO) Biological Process annotations. ORA was conducted with the enrichGO function implemented in the clusterProfiler R package (v4.16.0), applied to differentially expressed genes or predefined gene sets. The background set was defined as all genes detected in either bulk or single-cell RNA-seq datasets. Enriched GO categories were determined according to Benjamini-Hochberg-adjusted *p*-values. For GSEA, the fgsea R package (v1.34.2) was employed. Genes were ordered based on differential expression statistics, and enrichment significance was estimated through permutation testing with 100,000 iterations (nperm = 100,000).

### Network analysis

Protein-protein interaction (PPI) data were retrieved from the STRING database for mouse (v12.0). Interactions among differentially expressed genes were restricted to high-confidence pairs (combined score > 930) and used to assemble a network with the igraph R package (v2.1.4). Network communities were detected using the Walktrap method. To incorporate regulatory context, transcription factor (TF)-target relationships curated in the TRRUST v2 database were mapped onto the PPI network to infer upstream regulators. Subnetworks associated with thermogenesis were defined based on Gene Ontology (GO) Biological Process annotations. Within these subnetworks, node prioritization was performed using a Random Walk with Restart (RWR) approach. Functional characterization of individual modules was subsequently carried out using over-representation analysis implemented in enrichGO and enrichKEGG, and significance was evaluated after Benjamini-Hochberg correction. Network visualization was performed in Cytoscape through the RCy3 interface.

### Single-cell RNA-seq analysis

Single-cell RNA-seq datasets were analyzed using the Seurat R package (v5.3.0). Cells of low quality were excluded based on thresholds for nFeature_RNA, nCount_RNA, and the proportion of mitochondrial gene expression. Following quality control, the data underwent normalization and scaling, and batch effects were corrected through Harmony-based integration. Cell-type-specific marker genes were detected using the FindAllMarkers function, and cell identities were assigned according to established canonical markers. Hepatocyte populations were subsequently isolated and subjected to pseudobulk aggregation, where raw counts were combined at the sample level. The aggregated data were then analyzed with DESeq2, and differentially expressed genes were defined based on Benjamini-Hochberg-adjusted *p*-values.

### Mice

Seven-week-old male C57BL/6J mice were obtained from Hyochang Science (Daegu, Republic of Korea) and housed in groups under a 12-h light/dark cycle with ad libitum access to standard chow. *Cxcl5*-deficient mice were generated through deletion of exon 1 of the murine *Cxcl5* gene, as previously reported 15.

For diet-induced MASH studies, male mice at eight weeks of age were assigned to either an AMLN diet (40% kcal derived from fat, supplemented with fructose and cholesterol; #D09100310, Research Diets, New Brunswick, NJ, USA) or a control chow diet (10% kcal from fat) and maintained for the specified experimental periods.

In the adenoviral overexpression experiments, eight-week-old male C57BL/6J mice were fed a HFD (60% kcal from fat, #D12492, Research Diets) or standard chow (10% kcal from fat) for 12 weeks. Subsequently, mice received tail vein injections of 1 × 10⁹ plaque-forming units of either Ad-*Gfp* or Ad-*Cxcl5* (#000541A or #A417177; Applied Biological Materials, Richmond, Canada). After viral administration, animals continued on their respective diets for an additional 4 weeks before being euthanized for downstream analyses.

All animal procedures were conducted in compliance with the National Institutes of Health Guidelines for the Care and Use of Laboratory Animals and were approved by the Institutional Animal Care and Use Committee of Pusan National University (PNU-2022-0209).

### Measurement of hepatic non-esterified fatty acid (NEFA) content

Liver samples (~50 mg) were frozen, finely ground, and subsequently homogenized in ice-cold chloroform:methanol (2:1, v/v). Lipid extraction was performed using the classical Folch procedure 16. The concentration of NEFAs in liver extracts was quantified by a colorimetric assay using a commercial NEFA kit (#299-94301; Wako, Osaka, Japan) in accordance with the manufacturer's protocol 17.

### Measurement of hepatic triglyceride content

Triglyceride levels in liver tissues were quantified using a colorimetric assay kit (Cayman Chemical, Ann Arbor, MI, USA). Briefly, ~50 mg of frozen liver samples were finely ground and processed in accordance with the manufacturer's instructions.

### Measurement of serum alanine aminotransferase (ALT) and aspartate aminotransferase (AST)

Blood was collected from mice via the retro-orbital plexus, and serum was isolated for biochemical analysis. Activities of ALT and AST were measured using a Dri-Chem NX500 automated clinical chemistry analyzer (FujiFilm, Tokyo, Japan) in accordance with the manufacturer's instructions.

### Histological and immunohistochemical analysis

Mouse liver tissues were fixed in formalin, embedded in paraffin, and sectioned at a thickness of 4 μm. The sections were subjected to hematoxylin and eosin (H&E) and Sirius Red staining for histological evaluation. For immunohistochemistry, paraffin sections underwent heat-mediated antigen retrieval, followed by treatment with 3% H₂O₂ to quench endogenous peroxidase activity and blocking with 3% normal serum. Sections were then incubated with primary antibodies at 4 °C overnight. Signal detection was carried out using the Vectastain Elite ABC Kit and DAB Peroxidase Substrate Kit (Vector Laboratories, Burlingame, CA, USA) according to the manufacturer's guidelines. Primary antibodies against F4/80 (#70076) and α-smooth muscle actin (α-SMA; #19245) were obtained from Cell Signaling Technology (Danvers, MA, USA), while the MPO antibody (#PP023AA) was purchased from Biocare Medical (Concord, CA, USA). Bright-field images were captured using an AxioLab 5 microscope (Zeiss, Oberkochen, Germany). Quantification of positive staining was performed by analyzing both positive cell counts and stained areas across 10 randomly selected high-power fields using ImageJ software (National Institutes of Health, Bethesda, MD, USA).

### Cell culture

HepG2 cells (ATCC, Manassas, VA, USA) were maintained at 37 °C in a humidified atmosphere containing 5% CO₂ using Dulbecco's modified Eagle's medium (DMEM; #SH30243.01; Cytiva, Marlborough, MA, USA) supplemented with 10% fetal bovine serum (FBS; #SH30919.03; Cytiva) and 1% penicillin-streptomycin (#LS202-02; Welgene). AML12 cells (ATCC) were cultured under identical temperature and CO₂ conditions in a 1:1 mixture of DMEM and Ham's F12 medium (#LM002-08; Welgene, Gyeongsan, Republic of Korea), supplemented with insulin-transferrin-selenium (#41400045; Thermo Fisher Scientific, Waltham, MA, USA), 40 ng/mL dexamethasone (#D1961; Tokyo Chemical Industry, Tokyo, Japan), 10% FBS, and 1% penicillin-streptomycin. Cells were expanded in T-75 culture flasks and subsequently seeded into 6-, 12-, or 96-well plates for experimental procedures. Cultures were maintained at approximately 80% confluence and routinely passaged at least twice per week.

### Isolation of primary mouse hepatocytes

Primary hepatocytes were obtained from C57BL/6J mice, *Cxcl5*-deficient mice, and corresponding wild-type littermates using a standard two-step collagenase perfusion method 18. In brief, mice were anesthetized and the liver was perfused through the portal vein with calcium- and magnesium-free Hank's balanced salt solution (#LB003-04; Welgene, Gyeongsan, Republic of Korea) to clear residual blood, followed by perfusion with a collagenase-containing digestion buffer. Excised livers were gently dissociated to release hepatocytes, and the resulting suspension was passed through a 70-μm mesh filter. Hepatocytes were then enriched by low-speed centrifugation (50 × g, 5 min) and washed twice with Dulbecco's modified Eagle's medium (DMEM). Cells were subsequently maintained at 37 °C in a 5% CO₂ atmosphere in DMEM (#SH30243.01; Cytiva, Marlborough, MA, USA) supplemented with 10% FBS (#SH30919.03; Cytiva) and 1% penicillin-streptomycin (#LS202-02; Welgene).

### Conjugation of palmitic acid (PA) with bovine serum albumin (BSA)

Fatty acid-free BSA was obtained from GenDepot (Baker, TX, USA), and PA was purchased from Sigma-Aldrich (St. Louis, MO, USA). A PA stock solution (75 mM in ethanol) and a 5% (w/v) BSA solution prepared in culture medium were warmed to 60 °C prior to use. The PA solution was gradually introduced into the preheated BSA solution to achieve final concentrations of 5 mM PA and 5% BSA (equivalent to 0.8 mM), resulting in an approximate 6:1 molar ratio of PA to BSA. The mixture was then subjected to sonication until a clear solution was obtained, indicating complete dissolution 19.

### LPIN1 knockdown

HepG2 cells were subjected to siRNA-mediated knockdown targeting *LPIN1* using 50 pmol of specific siRNAs (catalog no. 4390771, assay no. s66131; Thermo Fisher Scientific, Waltham, MA, USA). A siRNA directed against green fluorescent protein (#SP-2011; Bioneer, Daejeon, Republic of Korea) served as a negative control. Transfections were carried out with Lipofectamine RNAiMAX reagent (#13778075; Thermo Fisher Scientific) in accordance with the manufacturer's instructions.

To investigate the functional role of LPIN1 *in vivo*, seven-week-old male *Cxcl5*-deficient mice were fed an AMLN diet for a total duration of 16 weeks. After initial 10 weeks of diet feeding, mice were randomly assigned to receive a single tail vein injection of an adeno-associated virus serotype 8 (AAV8) expressing either a short hairpin RNA (shRNA) targeting *Lpin1* (sh*Lpin1;* Vector Biolabs, Malvern, PA, USA) or a control shRNA against green fluorescent protein (sh*Gfp;* Vector Biolabs). The viruses were administered at a dose of 2 × 10^11^ genome copies (GC) per mouse. Following the injection, mice were maintained on an AMLN diet for an additional 6 weeks to allow sufficient gene knockdown and disease progression before sacrifice for further analysis.

### Plasmid construction and transient transfection

A plasmid encoding the full-length human *LPIN1* coding sequence was obtained from Dr. David Sabatini (#32005; Addgene, Watertown, MA, USA) 6. HepG2 cells were transiently transfected with the plasmid using Lipofectamine 3000 reagent (#L3000015; Thermo Fisher Scientific, Waltham, MA, USA) according to the manufacturer's instructions.

### Luciferase assay

A luciferase reporter construct containing the proximal promoter region (-421 bp) of the mouse *Lpin1* gene was generated (VectorBuilder, Chicago, IL, USA), based on prior identification of a functional glucocorticoid response element (GRE) within this promoter 20. A corresponding mutant reporter was created by substituting the last four nucleotides of the GRE motif with cytosine residues.

AML12 cells were co-transfected with the firefly luciferase reporter constructs and a thymidine kinase (TK)-driven Renilla luciferase plasmid for normalization. Transfection was carried out using Lipofectamine 3000 reagent (#L3000015; Thermo Fisher Scientific, Waltham, MA, USA) in accordance with the manufacturer's instructions.

Luciferase activities were determined in cell lysates using the Dual-Glo Luciferase Assay System (#E2940; Promega, Madison, WI, USA), and luminescence signals were measured with a microplate reader (Tecan, Männedorf, Switzerland). Firefly luciferase activity was normalized to Renilla luciferase activity.

### RNA isolation and real-time quantitative polymerase chain reaction (RT-qPCR)

Total RNA was extracted from cultured cells and tissue samples using RiboEx reagent (#301-002; Geneall, Seoul, Republic of Korea) following the manufacturer's protocol. Complementary DNA (cDNA) was synthesized from 1 μg of RNA using the ReverTraAce cDNA Synthesis Kit (#FSQ-101; Toyobo, Osaka, Japan). Quantitative PCR was performed with a SYBR Green-based detection system (#RT500M; Enzynomics, Daejeon, Republic of Korea) on a CFX Connect Real-Time PCR instrument (Bio-Rad, Hercules, CA, USA). Relative gene expression levels were calculated using the 2^-ΔΔCt^ method, with *Gapdh* and *Apob* serving as internal reference genes. Primer sequences are provided in Table S1.

### Cell lysis, subcellular fractionation, and immunoblot analysis

Cell and liver tissue samples were lysed using radioimmunoprecipitation assay (RIPA) buffer (#EBR001-500; Enzynomics, Daejeon, Republic of Korea) supplemented with protease and phosphatase inhibitor cocktails (#P3300-001; GenDepot, Baker, TX, USA), following the manufacturer's guidelines. Subcellular fractionation was carried out by differential centrifugation as described previously 21, 22. Protein concentrations were quantified using a bicinchoninic acid (BCA) assay kit (#23225; Thermo Fisher Scientific, Waltham, MA, USA).

For immunoblot analysis, protein samples were separated on 6-12% SDS-polyacrylamide gels and subsequently transferred onto nitrocellulose membranes (#10600003; Cytiva, Marlborough, MA, USA) 23. Membranes were developed using the Pierce ECL Western Blotting Substrate (#34580; Thermo Fisher Scientific), and signal detection was performed with a ChemiDoc MP Imaging System (Bio-Rad, Hercules, CA, USA).

Primary antibodies used in this study included β-actin (#sc-47778; Santa Cruz Biotechnology, Dallas, TX, USA), 4-hydroxynonenal (HNE; #MAB3249-SP; R&D Systems, Minneapolis, MN, USA), and PPAR-α (#NB600-636; Novus Biologicals, Littleton, CO, USA). Additional antibodies targeting phospho-p65 (#3033), p65 (#8242), phospho-GR (#97285), GR (#14041), phospho-p38 (#9216), p38 (#8690), phospho-JNK (#4668), JNK (#9252), LPIN1 (#14906), Lamin A/C (#4777), histone H3 (#4499), and α-tubulin (#2144) were obtained from Cell Signaling Technology (Danvers, MA, USA).

### Enzyme-linked immunosorbent assay (ELISA)

Levels of mouse CXCL5 in serum samples and cell culture supernatants were measured using a commercial Mouse LIX/CXCL5 ELISA kit (#KE10070; Proteintech, Rosemont, IL, USA) following the manufacturer's instructions.

### Neutrophil isolation and chemotaxis assay

Bone marrow cells were harvested from mouse femurs and tibias and filtered through a 70-μm mesh in phosphate-buffered saline (PBS). Following centrifugation at 300 × g for 5 min, the cell pellet was resuspended in ACK lysis buffer to remove erythrocytes. After a brief incubation on ice (2 min), cells were washed with PBS. Neutrophils were subsequently purified from the leukocyte fraction using a Mouse Neutrophil Isolation Kit (#130-097-658; Miltenyi Biotec, San Diego, CA, USA) according to the manufacturer's protocol.

For migration assays, AML12 cells were plated in the lower chamber of a 6-well plate and grown to approximately 80% confluence. Isolated neutrophils (1 × 10⁶ cells) were placed in transwell inserts (3-μm pore size) containing RPMI1640 medium supplemented with 10% FBS and 1% penicillin-streptomycin. To evaluate chemotactic responses, AML12 cells were stimulated with recombinant mouse CXCL5 (#250-17; Thermo Fisher Scientific, Waltham, MA, USA).

Cells that migrated to the lower chamber were collected from the medium and quantified using a hemocytometer after low-speed centrifugation to exclude residual AML12 cells.

### Statistical analysis

All transcriptomic analyses, including differential expression and subsequent bioinformatic procedures for both bulk and single-cell RNA-seq datasets, were carried out using R software (v4.5.0). Quantitative data are presented as mean ± standard error of the mean (SEM) and were analyzed using GraphPad Prism (GraphPad Software, La Jolla, CA, USA).

Comparisons between two groups were performed using Student's *t*-test, whereas differences among multiple groups were evaluated by one-way analysis of variance (ANOVA) followed by Tukey's post hoc test. A *p*-value < 0.05 was considered to indicate statistical significance.

## Results

### Hepatic *CXCL6* expression is elevated in patients with MASH

**Figure 1:**
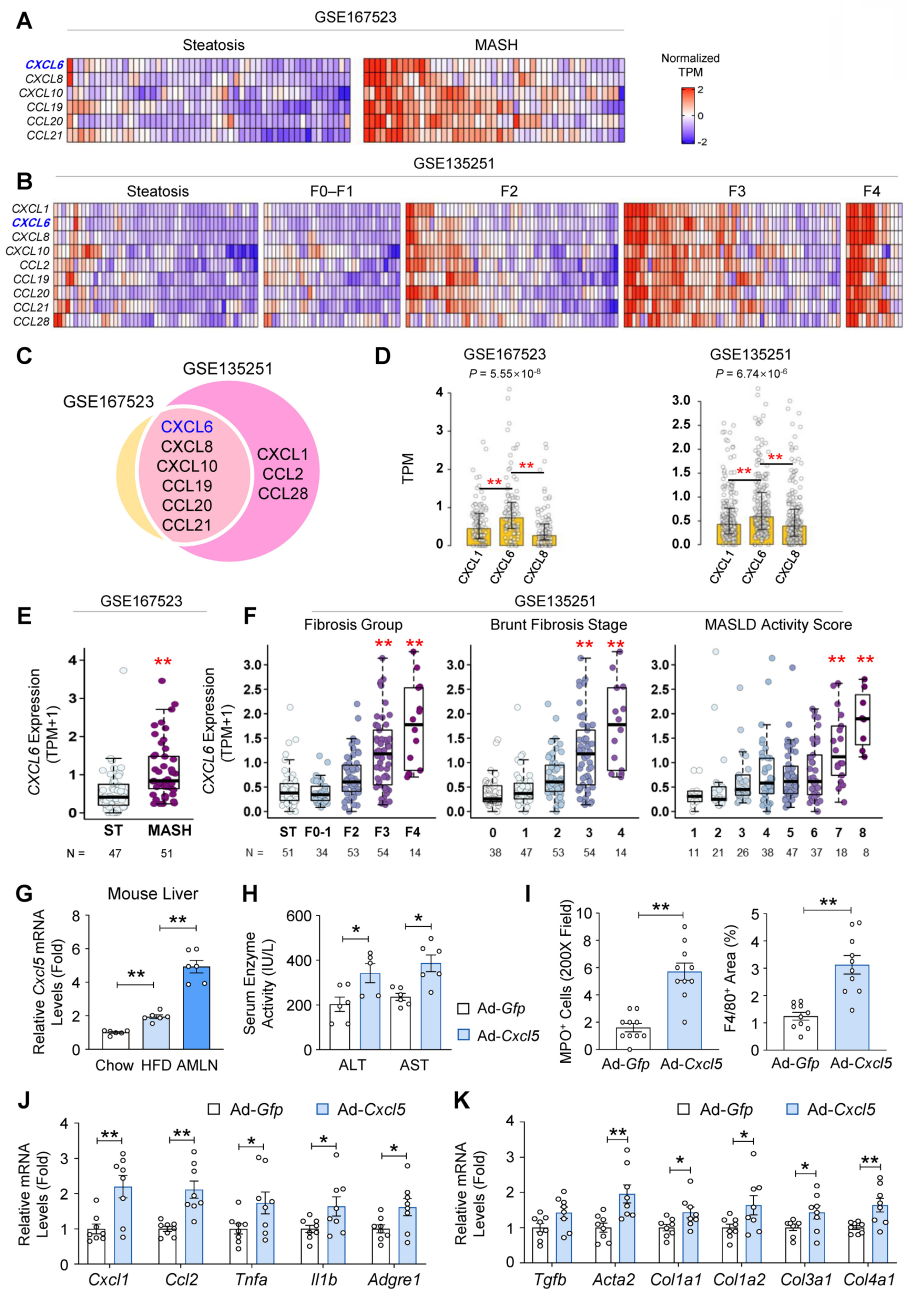
Hepatic overexpression of mouse *Cxcl5*, the homolog of human *CXCL6* upregulated in MASH, drives the progression from steatosis to MASH in HFD-fed mice. (A, B) Heatmap showing z-score-normalized TPM values of differentially expressed chemokine genes in the liver tissues of patients. Each column represents an individual patient, and each row corresponds to a chemokine gene. Chemokines significantly upregulated in MASH compared with steatosis (adjusted *p* < 0.01; log_2_FC > 1) are displayed. (C) Venn diagram depicting the overlap of chemokine genes that were significantly upregulated (adjusted *p* < 0.01; log_2_FC > 1) between GSE167523 (yellow) and GSE135251 (pink) datasets. (D) mRNA expression levels of *CXCL1*, *CXCL6*, and *CXCL8* in samples from patients with MASLD from both datasets. Bars indicate median TPM values, and error bars represent the interquartile range (Q1-Q3). Statistical significance was determined using the Kruskal-Wallis test followed by post-hoc pairwise comparisons (ns: not significant, *p < 0.05, **p < 0.01). (E) mRNA expression levels of the *CXCL6* gene in the liver of patients with steatosis (ST, *n* = 47) and MASH (*n* = 51) from a publicly available RNA sequencing database (GSE167523). Statistical significance was assessed using Student's *t*-tests (**p < 0.01). (F) mRNA expression levels of the *CXCL6* gene in the liver at different stages of MASLD, obtained from a publicly available RNA sequencing database (GSE135251). F0-F1, F2, F3, and F4 represent different MASH groups classified by the severity of fibrosis. TPM refers to transcripts per million base pairs. For comparisons involving more than two groups, one-way analysis of variance (ANOVA) was conducted, followed by post-hoc Tukey's tests to determine specific group differences (**p < 0.01). (G) Male C57BL/6J mice (*n* = 6) were fed a chow diet (12 weeks), a high-fat diet (HFD, 12 weeks), and a gubra amylin (AMLN) diet (16 weeks). Hepatic *Cxcl5* mRNA levels were determined using RT-qPCR. (H-K) Male C57BL/6J mice (*n* = 6) were fed a high-fat diet for 16 weeks. At week 12, the mice were injected with an adenovirus expressing *Cxcl5* (Ad-*Cxcl5*) or *Gfp* (Ad-*Gfp*) as a control. At week 16, the mice were sacrificed, and blood and liver tissues were collected for analysis. (H) Serum ALT and AST levels. (I) Paraffin-embedded liver tissue was stained for myeloperoxidase (MPO) and F4/80. The number of MPO-positive cells per 200X field and the area positive for F4/80 staining were quantified (right). The representative images used for quantification are shown in Fig. S3F. (J, K) Liver tissues were subjected to RT-qPCR analysis for proinflammatory (panel J) and fibrogenic genes (panel K). Values represent mean ± SEM. Statistical significance was assessed using Student's *t*-tests (*p < 0.05, **p < 0.01).

To identify the key chemokines implicated in MASH pathogenesis, we examined the expression profiles of chemokine genes using two publicly available RNA-seq datasets (GSE167523 and GSE135251). Analysis of GSE167523 showed that *CXCL6, CXCL8, CXCL10, CCL19, CCL20*, and *CCL21* were significantly upregulated in liver tissues from patients with MASH compared with those from patients with steatosis (adjusted *p*-value < 0.01; log_2_FC > 1) (Fig. 1A). In the GSE135251 dataset, *CXCL1*, *CXCL6, CXCL8, CXCL10, CCL2, CCL19, CCL20, CCL21, and CCL28* were significantly increased in MASH (F0-F4) compared with steatosis (Fig. 1B). Cross-comparison of both datasets identified *CXCL6, CXCL8, CXCL10, CCL19, CCL20,* and* CCL21* as commonly upregulated genes (Fig. 1C). Among these, neutrophil chemotaxis is stimulated by *CXCL6* and* CXCL8*. Hepatic *CXCL6* expression exceeded the expression of major neutrophil-recruiting chemokines such as *CXCL1* and *CXCL8* during MASLD progression (Fig. 1D and Fig. S1). The analysis of GSE167523 confirmed that *CXCL6* expression was higher in patients with MASH than in those with simple steatosis (Fig. 1E). The analysis of GSE135251 demonstrated that *CXCL6* expression increased proportionally with disease severity, as defined by the fibrosis group, Brunt fibrosis stage, and MASLD activity score (Fig. 1F). This reinforces the close association of CXCL6 with MASLD progression.

### *Cxcl5* overexpression promotes the progression of steatosis to MASH in HFD-fed mice

Next, we examined the expression of *Cxcl5*, the murine homolog for the human *CXCL6* gene, in the livers of mice fed a chow diet, HFD (inducing steatosis), or AMLN diet (inducing MASH). Hepatic *Cxcl5* expression increased in HFD-fed mice and was further elevated in AMLN diet-fed mice (Fig. 1G). Serum CXCL5 protein levels were elevated in AMLN diet-fed mice (Fig. S2). These observations led us to hypothesize that hepatic overexpression of *Cxcl5* may promote the progression of steatosis to MASH in HFD-fed obese mice. To test this, mice were fed a HFD for 12 weeks to induce steatosis, followed by tail vein injections of adenovirus expressing mouse *Cxcl5* (Ad-*Cxcl5*) or *Gfp* (Ad-*Gfp*) as a control (Fig. S3A). Ad-*Cxcl5* caused *Cxcl5* overexpression predominantly in the liver (Fig. S3B). Serum CXCL5 protein levels were also elevated by Ad-*Cxcl5* infection (Fig. S3C). Hepatocyte *Cxcl5* overexpression was confirmed by the increased secretion of CXCL5 protein from primary hepatocytes isolated from Ad-*Cxcl5*-injected mice (Fig. S3D). *Cxcl5* overexpression exacerbated liver injury, as evidenced by the elevated serum levels of ALT and AST (Fig. 1H), without affecting body or liver weight (Fig. S3E). *Cxcl5* overexpression promoted liver inflammation as indicated by an increased population of neutrophils and macrophages in the liver (Fig. 1I and Fig. S3F). Hepatic *Cxcl5* overexpression induced the expression of inflammatory and fibrogenic genes (Figs. 1J-1K). These findings suggest that hepatic *Cxcl5* overexpression promotes the progression from steatosis to MASH in HFD-fed mice.

### *Cxcl5* deletion reduces hepatic injury and neutrophil infiltration in AMLN diet-fed mice

**Figure 2:**
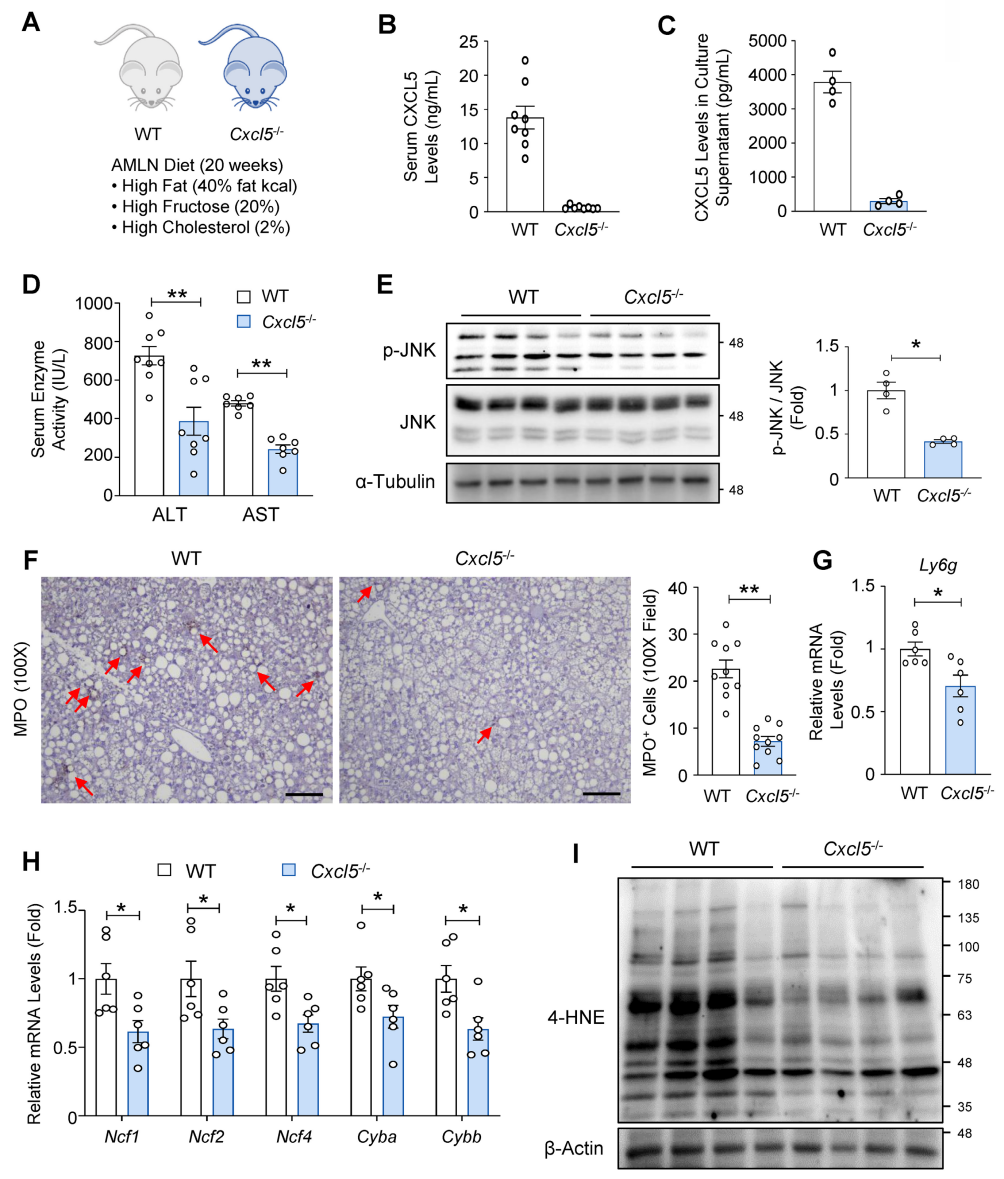
***Cxcl5* knockout ameliorates liver injury in AMLN diet-fed mice.** (A) *Cxcl5*-deficient mice and wild-type (WT) littermate controls were fed an AMLN diet for 20 weeks to induce MASH (*n* = 7). (B) Serum CXCL5 levels were examined using ELISA. (C) Primary hepatocytes isolated from *Cxcl5*-deficient mice and WT littermate controls were cultured for 24 h. Culture supernatants were obtained and subjected to ELISA analysis of CXCL5. (D) Serum ALT and AST levels were assessed. (E) Liver tissues were subjected to immunoblot analysis of phosphorylated JNK (p-JNK) and JNK (left). Relative levels of p-JNK were quantified after normalization to JNK (right). (F) Paraffin-embedded liver tissues were subjected to MPO staining (left). Red arrowheads indicate MPO-positive cells. The number of MPO-positive cells per 100X field was counted (right). Scale bars indicate 200 μm. (G) Hepatic *Ly6g* mRNA levels were assessed using RT-qPCR. (H) Hepatic mRNA levels of NADPH oxidase 2 (NOX2) complex components were assessed using RT-qPCR. (I) Liver tissues were subjected to immunoblot analysis of 4-HNE. Values represent mean ± SEM. Statistical significance was assessed using Student's *t*-tests (*p < 0.05, **p < 0.01).

Given the MASH-promoting effects of hepatic *Cxcl5* overexpression, we investigated the functional role of *Cxcl5* in MASH pathogenesis using *Cxcl5* knockout mice. *Cxcl5* knockout mice and wild-type littermate controls were subjected to 20-week feeding with an AMLN diet to induce MASH (Fig. 2A). CXCL5 levels were reduced in the serum of *Cxcl5*-deficient mice (Fig. 2B) and in the culture supernatants of primary hepatocytes isolated from *Cxcl5*-deficient mice (Fig. 2C), confirming effective hepatic deletion. Serum CXCL1 levels were lower than CXCL5 levels in wild-type mice, indicating a relatively abundant expression of *Cxcl5* compared with *Cxcl1* in mice (Fig. S4A). Serum CXCL1 levels were not altered by *Cxcl5* deletion (Fig. S4A). Body, liver, and epididymal fat weights were not changed by *Cxcl5* deletion (Fig. S4B). *Cxcl5* deficiency ameliorated AMLN diet-induced liver injury, as shown by the reduction in serum ALT and AST levels (Fig. 2D). The activation of the stress kinase JNK was attenuated in the livers of *Cxcl5*-deficient mice (Fig. 2E). These results indicate that *Cxcl5* deletion ameliorated AMLN diet-induced liver injury in mice. Since CXCL5 is a neutrophil chemoattractant in mice, we examined neutrophil infiltration in the liver. Hepatic neutrophil infiltration was attenuated in *Cxcl5* knockout mice (Fig. 2F and Fig. S4C). Similarly, the hepatic mRNA levels of *Ly6g*, a neutrophil marker, were reduced in *Cxcl5*-deficient mice (Fig. 2G). We have previously reported that neutrophils contribute to MASH pathogenesis by upregulating NADPH oxidase 2 (NOX2) complex components, which mediate neutrophil oxidative bursts 9. Consistent with this, *Cxcl5* deficiency lowered the hepatic mRNA levels of NOX2 complex components (Fig. 2H) and decreased hepatic 4-HNE adduct formation, a marker of oxidative stress (Fig. 2I).

### *Cxcl5* deletion reduces hepatic inflammation and fibrosis in AMLN diet-fed mice

**Figure 3:**
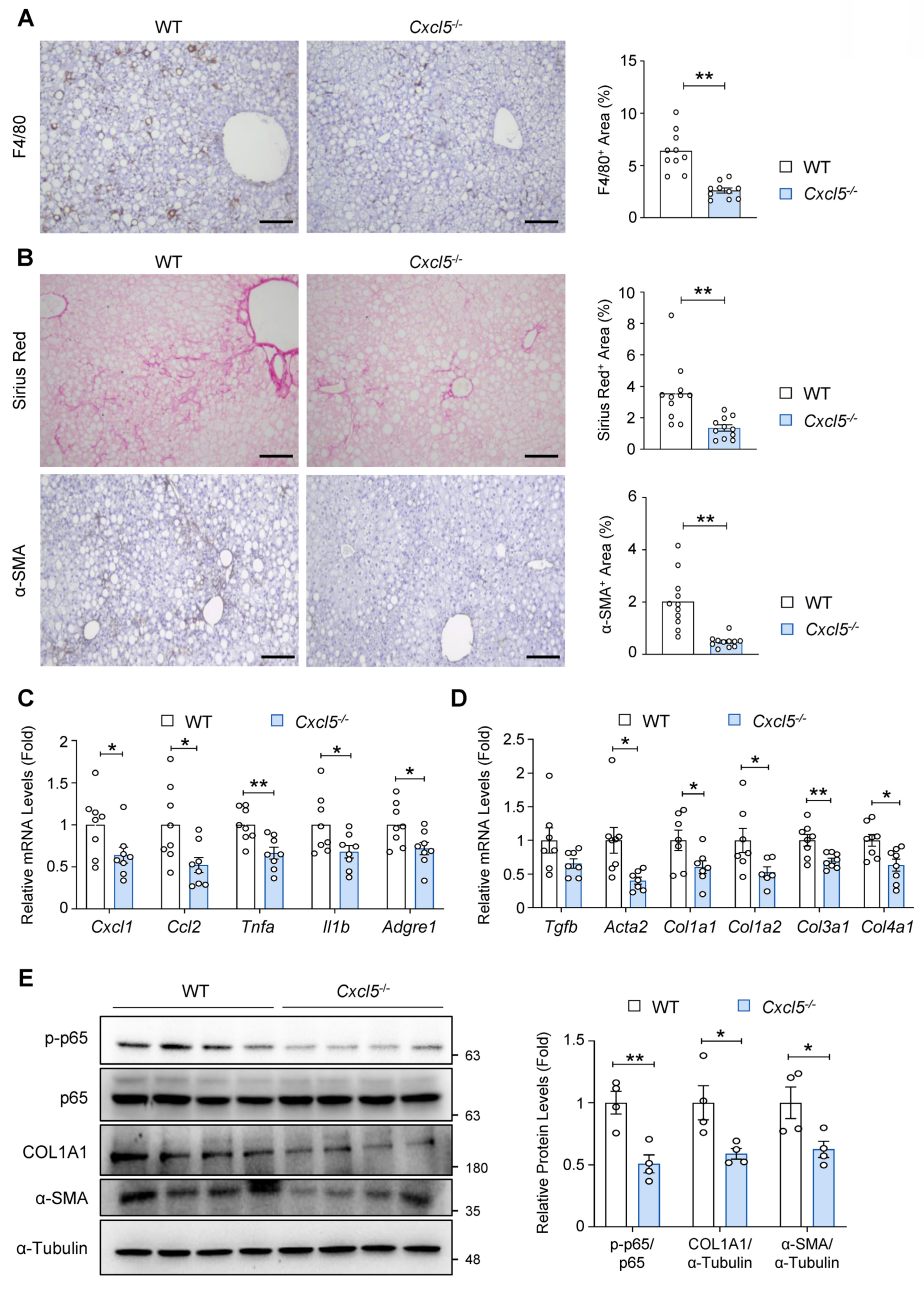
***Cxcl5* knockout ameliorates liver inflammation and fibrosis in AMLN diet-fed mice.** *Cxcl5*-deficient mice and wild-type (WT) littermates were fed an AMLN diet for 20 weeks to induce MASH (*n* = 7). (A) Paraffin-embedded liver tissue was subjected to F4/80 staining (left). The area positive for F4/80 staining was quantified (right). (B) Paraffin-embedded liver tissues were subjected to Sirius Red (top) and α-SMA staining (bottom). The area positive for each stain was quantified (right). Scale bars indicate 200 μm. (C, D) Liver tissues were subjected to RT-qPCR analysis of proinflammatory (panel C) and fibrogenic genes (panel D). (E) Liver tissues were subjected to immunoblot analysis of phosphorylated p65 (p-p65), p65, COL1A1, and α-SMA (left). Relative expression of p-p65 normalized to p65, COL1A1 normalized to α-Tubulin, and α-SMA normalized to α-Tubulin was quantified (right). Values represent mean ± SEM. Statistical significance was assessed using Student's *t*-tests (*p < 0.05, **p < 0.01).

To further assess the role of *Cxcl5* in MASH pathogenesis, we examined whether *Cxcl5* deletion attenuates hepatic inflammation and fibrosis, two key features of advanced MASLD, in AMLN diet-fed mice. Immunohistochemical analysis of F4/80 indicated reduced hepatic macrophage infiltration in *Cxcl5*-deficient mice compared with that in wild-type controls (Fig. 3A). Collagen deposition and myofibroblast formation were reduced in *Cxcl5* knockout mice, indicating attenuated liver fibrosis by *Cxcl5* ablation (Fig. 3B and Fig. S4D). Similarly, RT-qPCR analysis demonstrated that *Cxcl5* deletion reduced the mRNA levels of proinflammatory genes, such as *Cxcl1*, *Ccl2*, *Tnfa*, *Il1b*, and *Adgre1* (Fig. 3C). The expression of fibrogenic genes also decreased in the livers of *Cxcl5*-deficient mice (Fig. 3D). Immunoblot analysis showed that p65 phosphorylation was inhibited by *Cxcl5* deletion in the liver, and fibrosis-related factors, such as COL1A1 and α-SMA, were downregulated in *Cxcl5*-deficient mice (Fig. 3E). Along with our gain-of-function data, these results suggest that *Cxcl5* may contribute to MASLD progression in mice.

### LPIN1, PPARα, and FA oxidation-related genes are upregulated in the liver of *Cxcl5*-deficient mice

**Figure 4:**
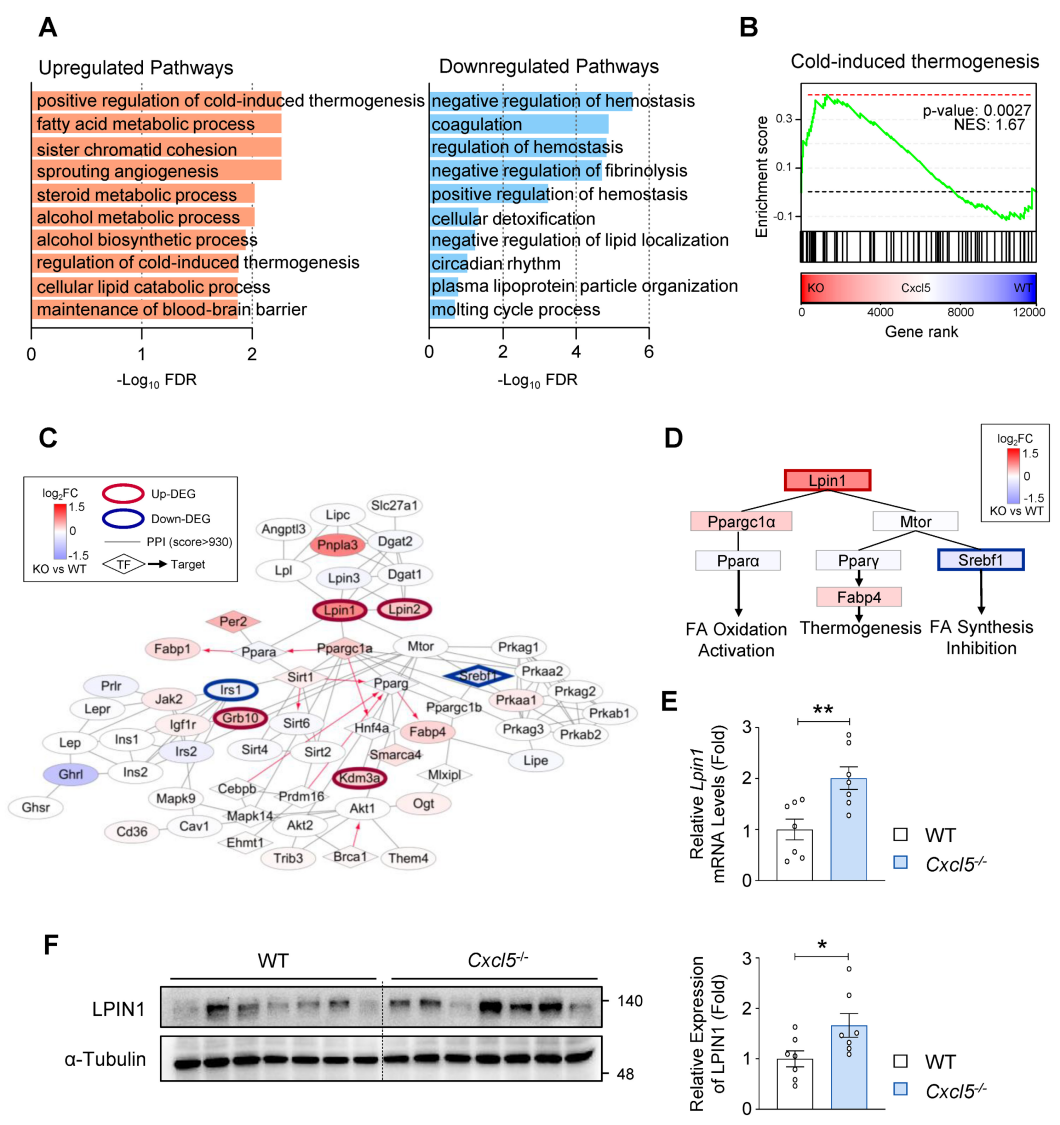
*Cxcl5* deficiency induces the expression of LPIN1 and upregulates genes involved in fatty acid metabolic processes in AMLN diet-fed mice. RNA-seq analysis was performed using the liver tissues of *Cxcl5*-deficient mice and wild-type (WT) littermate controls subjected to 20 weeks of AMLN diet feeding. (A) Bar plots showing the top 10 Gene Ontology (GO) Biological Process terms significantly enriched among differentially upregulated (left, orange) and downregulated (right, blue) genes *Cxcl5*^-/-^ livers relative to WT controls. (B) Gene set enrichment analysis (GSEA) demonstrating significant upregulation of the cold-induced thermogenesis gene set in *Cxcl5*^-/-^ livers compared with WT. (C) Subnetwork extracted from the protein-protein interaction network highlighting genes associated with thermogenesis and fatty acid metabolism. Node colors indicate expression changes (log_2_FC for *Cxcl5*^-/-^ vs. WT), and directed edges represent regulatory relationships; hexagonal nodes denote transcription factors (TFs). (D) Schematic representation of the regulatory network linking LPIN1 to PPARα, PPARγ, and SREBP1 signaling pathways. Node colors indicate expression changes (log_2_FC for *Cxcl5*^-/-^ vs. WT). (E) RT-qPCR analysis of *Lpin1* expression in liver tissues from *Cxcl5*-deficient mice and WT controls. (F) Liver tissues from *Cxcl5*-deficient mice and WT controls were subjected to immunoblotting for LPIN1 (left). Relative expression of LPIN1 normalized to α-Tubulin was quantified (right). Values represent mean ± SEM. Statistical significance was assessed using Student's *t*-tests (*p < 0.05, **p < 0.01).

To determine the molecular mechanisms by which *Cxcl5* influences MASLD progression in mice, we performed RNA-seq analysis of the livers from *Cxcl5*-deficient and wild-type mice. This analysis identified 149 upregulated and 202 downregulated genes in *Cxcl5*-deficient mice (Fig. S5A). Functional enrichment analysis showed that genes related to cold-induced thermogenesis and FA metabolism were significantly upregulated in *Cxcl5*-deficient mice (Fig. 4A). These two pathways are functionally interconnected because mitochondrial FA β-oxidation plays a critical role in thermogenesis, particularly in adipose tissue. Several thermogenesis-related genes also regulate FA metabolism in the liver, and both pathways included commonly upregulated genes, such as *Lpin1* and *Fabp5* (Fig. S5B). GSEA further confirmed the upregulation of thermogenesis-related genes in *Cxcl5* knockout mice (Fig. 4B). Further PPI network analysis using genes involved in thermogenesis and FA metabolism showed that *Lpin1*, one of the top upregulated genes, is centrally linked to *Ppara* (PPARα) and *Ppargc1a* (PGC1α), a coactivator of PPARα (Fig. 4C) Consistent with these findings, the network topology highlights four interconnected gene modules linking FA metabolism and cold-induced thermogenesis, positioning *Lpin1* at the center of this regulatory circuitry (Fig. S5C). LPIN1 activates PPARα and promotes the transcription of FA oxidation genes through a mechanism that requires PGC1α 4 (Fig. 4D). Since lipotoxicity resulting from excessive hepatic FA accumulation contributes to liver injury and inflammation in MASH, enhanced FA oxidation and reduced lipotoxicity may underlie the attenuated MASH phenotype observed in *Cxcl5-*deficient mice. Nuclear LPIN1 was shown to suppress SREBP-mediated FA synthesis through mTORC1 regulation 6. We observed reduced expression of *Srebf1* (encoding SREBP-1) in *Cxcl5* knockout mice (Fig. 4D). These transcriptomic findings were further validated by RT-qPCR and immunoblot analyses, which confirmed the elevated hepatic expression of *Lpin1* in *Cxcl5* knockout mice (Figs. 4E-4F). Taken together, these data suggest that *Cxcl5* deletion promotes hepatic FA metabolism and may attenuate hepatic lipotoxicity, and *Lpin1* could critically contribute to the beneficial changes observed in *Cxcl5* knockout mice.

### CXCL6 inhibits PPARα and FA oxidation-related genes in hepatocytes

**Figure 5:**
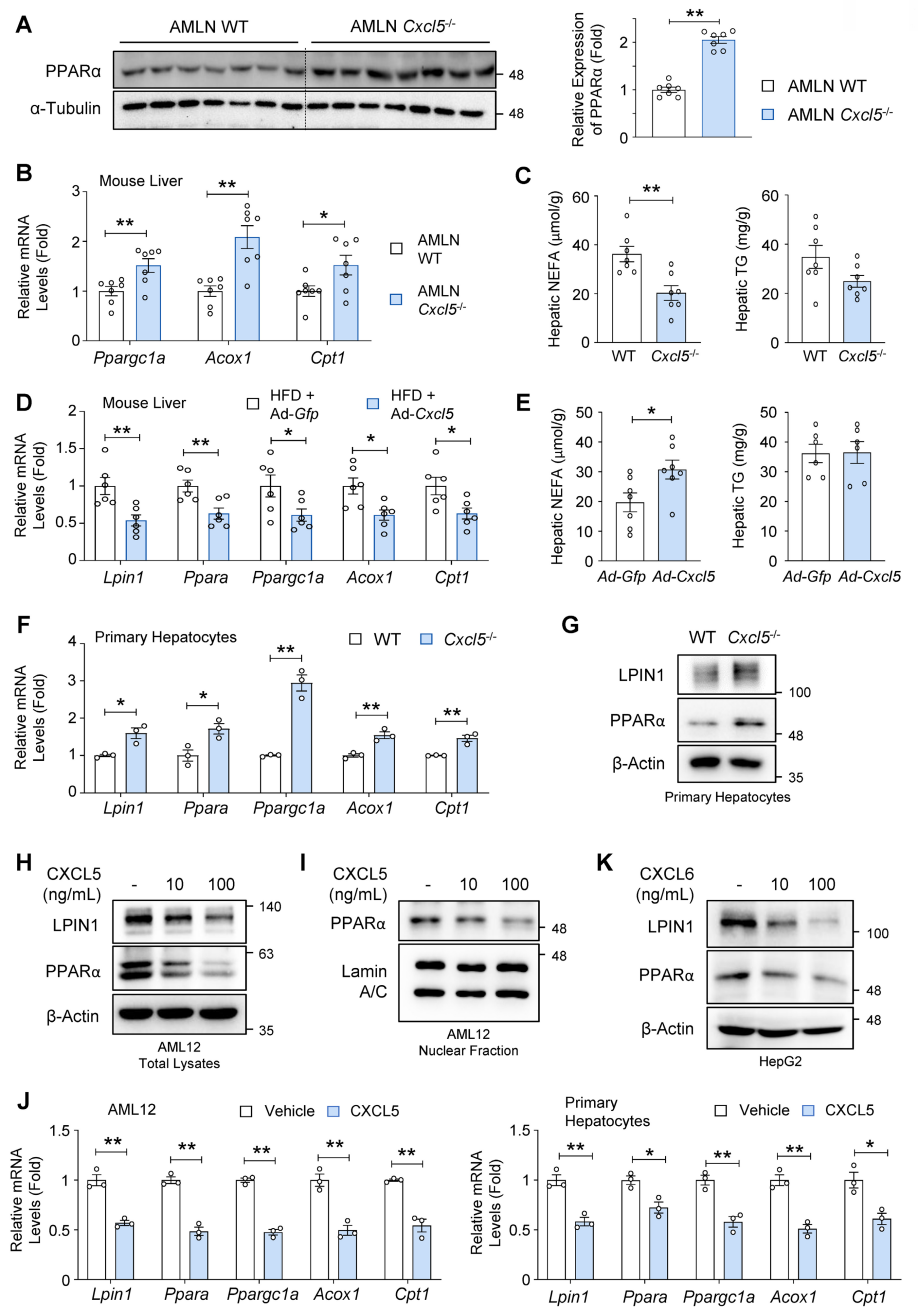
** CXCL5/6 inhibits LPIN1 and PPARα via direct effects on hepatocytes.** (A-C) *Cxcl5*-deficient mice and wild-type (WT) littermate controls were fed an AMLN diet for 20 weeks to induce MASH (*n* = 7). (A) Liver tissues of *Cxcl5*-deficient mice and WT littermate controls were subjected to immunoblot analysis of PPARα (left). Relative expression of PPARα normalized to α-Tubulin was quantified (right). (B) RT-qPCR analysis of hepatic *Ppargc1a*,* Acox1*, and* Cpt1*. (C) Hepatic levels of non-esterified fatty acids (NEFAs) and triglycerides (TGs). (D, E) Male C57BL/6J mice (*n* = 6) were fed a high-fat diet (HFD) for 16 weeks. At week 12, the mice were injected with an adenovirus expressing *Cxcl5* (Ad-*Cxcl5*) or *Gfp* (Ad-*Gfp*) as a control. At week 16, the mice were sacrificed, and blood and liver tissues were collected for analysis. (D) Liver tissues were subjected to RT-qPCR analysis of FA oxidation genes. (E) Hepatic levels of NEFAs and TGs. (F) Primary hepatocytes isolated from *Cxcl5*-deficient mice and WT littermate controls were subjected to RT-qPCR analysis of FA oxidation genes. (G) Primary hepatocytes isolated from *Cxcl5*-deficient mice and WT littermate controls were subjected to immunoblot analysis of LPIN1 and PPARα. (H, I) AML12 cells were treated with vehicle or recombinant mouse CXCL5 (10 or 100 ng/mL) for 24 h. Total cell lysates (panel H) and nuclear extracts (panel I) were subjected to immunoblot analysis. (J) AML12 cells and primary mouse hepatocytes were treated with vehicle or recombinant mouse CXCL5 (100 ng/mL) for 24 h. RT-qPCR analysis of FA oxidation genes was performed. (K) HepG2 cells were treated with vehicle or recombinant human CXCL6 (10 or 100 ng/mL) for 24 h. Total cell lysates were subjected to immunoblot analysis. Statistical significance was assessed using Student's t-test (*p < 0.05, **p < 0.01).

The observations that *Lpin1* was upregulated in *Cxcl5* knockout mice prompted us to examine the impact of *Cxcl5* deficiency on the expression of PPARα and FA oxidation-related genes. Hepatic PPARα expression was significantly elevated in *Cxcl5*-deficient mice (Fig. 5A). RT-qPCR analysis indicated that *Ppargc1a* and FA oxidation-related genes, such as *Acox1* and *Cpt1*, were upregulated in the livers of *Cxcl5*-deficient mice (Fig. 5B). In agreement with this, hepatic NEFA levels were reduced in *Cxcl5*-deficient mice, while hepatic triglyceride levels showed a non-significant trend toward reduction (Fig. 5C).

We examined whether hepatic *Cxcl5* overexpression exerted the opposite effects. To this end, we analyzed the identical samples used in the experiments presented in Figs. 1H-1K. *Cxcl5* overexpression reduced the mRNA levels of *Lpin1*, *Ppara*, *Ppargc1a*, *Acox1*, and *Cpt1* in the liver of HFD-fed mice (Fig. 5D). *Cxcl5* overexpression increased the hepatic NEFA levels in HFD-fed mice (Fig. 5E).

Analysis of single-cell RNA-seq data (GSE136103) demonstrated that human *CXCL6* is predominantly expressed by hepatocytes in the liver, and its expression is increased in patients with end-stage liver diseases, such as hepatic cirrhosis (Figs. S6A-S6D). Since hepatic FA oxidation mainly occurs in hepatocytes 24, we investigated the role of CXCL6 in the regulation of FA oxidation in hepatocytes. The expression levels of *Lpin1*, *Ppara*, *Ppargc1a*, and FA oxidation-related genes (e.g., *Acox1, Cpt1*) were higher in *Cxcl5*-deficient hepatocytes than in wild-type hepatocytes (Fig. 5F). At protein levels, *Cxcl5* deletion upregulated LPIN1 and PPARα in primary mouse hepatocytes (Fig. 5G). These results suggest that hepatocyte-derived CXCL6 may act on hepatocytes to inhibit LPIN1 and PPARα in a hepatocyte-intrinsic manner.

Treatment with recombinant murine CXCL5 inhibited the expression of LPIN1 and PPARα in a dose-dependent manner (Fig. 5H). Murine CXCL5 treatment reduced the expression of PPARα in the nucleus, where PPARα transactivates the genes involved in FA oxidation (Fig. 5I). Murine CXCL5 treatment consistently reduced the mRNA levels of *Lpin1*, *Ppara*, and FA oxidation-related genes in AML12 cells and primary mouse hepatocytes (Fig. 5J). The treatment with recombinant human CXCL6 inhibited the expression of LPIN1 and PPARα in HepG2 human hepatoma cells (Fig. 5K). Impaired FA oxidation was further supported by the reduced expression of mitochondrial genes essential for mitochondrial respiration and oxidative metabolism, including *mt-Co1*, *Ucp2*, *Cycs*, *Atp5f1b*, and *Tfam* (Fig. S7).

We hypothesized that FA loading induces CXCL6 expression in hepatocytes during MASH development, contributing to impaired FA oxidation and aggravated lipotoxicity. To test this hypothesis, AML12 and HepG2 cells were treated with PA. PA treatment increased the expression of multiple CXCR2 ligand genes in both AML12 and HepG2 cells. In AML12 (mouse) cells, *Cxcl5* showed greater induction than *Cxcl1* and *Cxcl2*, and CXCL5 protein levels were elevated in the culture supernatant (Figs. S8A-S8B). In HepG2 (human) cells, *CXCL6* exhibited greater induction than *CXCL1*, *CXCL2*, and *CXCL8* (Fig. S8C).

As CXCL6 stimulates neutrophil recruitment, and *Cxcl5* knockout reduced hepatic neutrophil population in mice (Fig. 2F), we investigated whether exposure to neutrophils affects the expression of LPIN1 and PPARα in hepatocytes. Treatment with recombinant mouse CXCL5 (100 ng/mL) led to the migration of approximately 200,000 neutrophils from an initial population of 1,000,000, compared with approximately 10,000 neutrophils treated with the vehicle (Fig. S9A). We then cultured AML12 cells in the conditioned media obtained from (1) 10,000 and (2) 200,000 neutrophils. Conditioned media from 200,000 neutrophils did not reduce the mRNA levels of *Lpin1, Ppara, Ppargc1a, Acox1,* or *Cpt1* in AML12 cells compared with the conditioned media from 10,000 neutrophils, indicating that the abundance of neutrophils in the hepatocyte milieu does not affect FA oxidation gene expression in hepatocytes (Fig. S9B). These findings suggest that lipotoxic hepatocytes induce CXCL6 upregulation. This may inhibit the expression of LPIN1 and PPARα-regulated FA oxidation genes via direct effects on hepatocytes, independently of neutrophil-mediated mechanisms.

### CXCL6 inhibits PPARα and FA oxidation-related genes via suppression of GR and LPIN1

**Figure 6:**
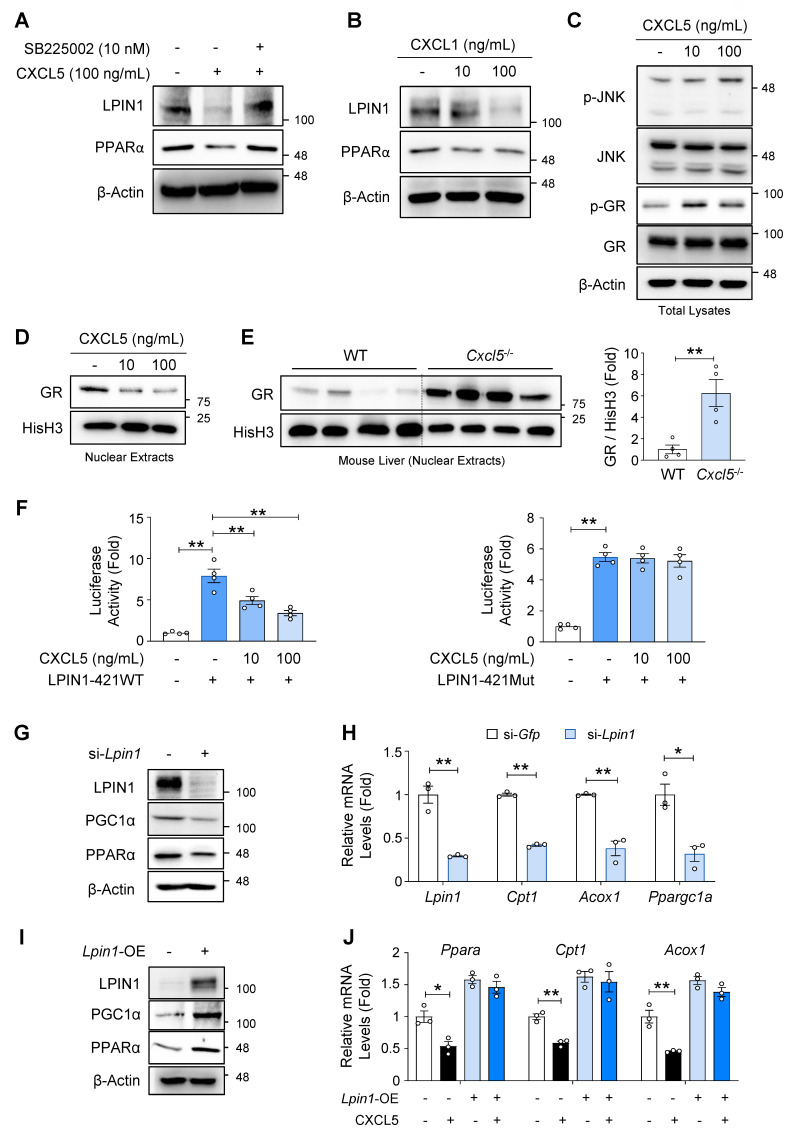
** CXCL5/6 suppresses PPARα and FA oxidation genes through GR phosphorylation and LPIN1 inhibition in hepatocytes.** (A) AML12 cells were treated with recombinant mouse CXCL5 (100 ng/mL) or vehicle following a 30-min pretreatment with SB225002 (10 nM) or vehicle. Immunoblot analysis of LPIN1 and PPARα was conducted. (B) AML12 cells were treated with recombinant mouse CXCL1 (10 or 100 ng/mL) or vehicle for 24 h. Immunoblot analysis of LPIN1 and PPARα was conducted. (C, D) AML12 cells were treated with recombinant mouse CXCL5 (10 or 100 ng/mL) or vehicle for 24 h. Total lysates (panel C) and nuclear extracts (panel D) of the treated cells were subjected to immunoblot analysis. (E) Nuclear extracts of the liver samples from *Cxcl5*-deficient mice and wild-type (WT) littermate controls fed an AMLN diet were subjected to immunoblot analysis of GR (left). Relative expression of GR normalized to histone H3 (His-H3) was quantified (right). (F) AML12 cells were cultured in media supplemented with dexamethasone (100 nM), an essential component for their growth, with insulin, transferrin, and selenium. The cells were transfected for 24 h with luciferase reporter constructs containing the mouse *Lpin1* promoter harboring a WT GRE (LPIN1-421WT) or a mutated GRE (LPIN1-421Mut) (Fig. S12). Concomitantly, the cells were treated with vehicle or recombinant mouse CXCL5 (10 or 100 ng/mL). Renilla luciferase plasmid was co-transfected for normalization. (G, H) AML12 cells were transfected with an siRNA targeting *Lpin1* (25 pmol) or *Gfp* as a control. (G) Total cell lysates were subjected to immunoblot analysis of LPIN1, PGC1α, and PPARα. (H) Transcript levels of *Lpin1, Cpt1, Acox1,* and *Ppargc1a* were assessed by RT-qPCR. (I) AML12 cells were transfected with a plasmid overexpressing mouse *Lpin1* (*Lpin1*-OE; 1 μg) or pcDNA3.1 as a control for 24 h. Total lysates were subjected to immunoblot analysis of LPIN1, PGC1α, and PPARα. (J) AML12 cells were transfected with *Lpin1*-OE (1 μg) or pcDNA3.1 as a control for 24 h. Concomitantly, cells were treated with recombinant mouse CXCL5 (100 ng/mL) or vehicle. Transcript levels of *Ppara, Cpt1,* and* Acox1* were assessed by RT-qPCR. Values represent mean ± SEM. Statistical evaluation was performed using one-way ANOVA with Tukey's post hoc test for multiple comparisons or Student's *t*-tests (*p < 0.05, **p < 0.01).

Having established that CXCL6 inhibits LPIN1, PPARα, and FA oxidation genes in hepatocytes, we investigated the mechanisms by which CXCL6 inhibits LPIN1. Because CXCL6 signals through the CXCR2 receptor, we determined whether the inhibitory effect of CXCL6 on LPIN1 and PPARα is mediated by CXCR2 activation. Treating AML12 cells with recombinant mouse CXCL5 reduced LPIN1 and PPARα expression. This effect was reversed by SB225002, a selective CXCR2 inhibitor (Fig. 6A), indicating that CXCL6 inhibits LPIN1 and PPARα via CXCR2 activation. *Cxcr2* mRNA levels showed a tendency to increase following CXCL5 exposure (Fig. S10). CXCL1, another CXCR2 ligand, also decreased the expression of LPIN1 and PPARα in AML12 cells, further supporting the involvement of CXCR2 (Fig. 6B).

Next, we explored how the CXCL6-CXCR2 pathway regulates LPIN1 expression. The glucocorticoid receptor (GR) is a nuclear receptor that transcriptionally regulates LPIN1 expression 20. JNK promotes GR phosphorylation at Ser226, which facilitates nuclear export of GR and reduces its transcriptional activity 25-29. Because CXCR2 activation triggers JNK activation 30, 31, we examined whether CXCL6 enhanced JNK and GR phosphorylation. Recombinant mouse CXCL5 increased JNK phosphorylation in AML12 cells, accompanied by elevated GR phosphorylation at Ser226 (Fig. 6C). Therefore, nuclear GR expression was decreased in AML12 cells (Fig. 6D). Nuclear GR expression was consistently elevated in the liver of *Cxcl5* knockout mice (Fig. 6E), which coincided with reduced JNK phosphorylation (Fig. 2E). The inhibitory effect of recombinant CXCL5 on LPIN1 expression was reversed by a pretreatment with SP600125, a JNK inhibitor (Fig. S11). Consistently, the ability of recombinant CXCL5 to suppress nuclear GR expression was attenuated by SP600125. These results indicate that CXCL6 inhibits GR and LPIN1 in a JNK-dependent manner. To determine whether GR inhibition functionally affects *Lpin1* transcription, we performed luciferase reporter assays using constructs containing the mouse *Lpin1* promoter. Based on the previously identified GRE within the *Lpin1* promoter 20, we used luciferase reporter constructs containing the mouse *Lpin1* promoter with wild-type GRE (LPIN1-421WT) or mutated GRE (LPIN1-421Mut) (Fig. S12). Recombinant mouse CXCL5 significantly suppressed luciferase activity driven by the wild-type promoter, but not by the mutated version (Fig. 6F). This confirmed that CXCL6 represses LPIN1 via GRE-dependent transcriptional repression. These results indicate that CXCL6 activates JNK and promotes the phosphorylation and nuclear export of GR, thereby suppressing LPIN1 expression.

To determine whether LPIN1 mediates the inhibitory effects of CXCL6 on PPARα and FA oxidation, we modulated the *Lpin1* expression in AML12 cells. *Lpin1* knockdown inhibited the expression of PGC1α and PPARα (Fig. 6G) and downregulated FA oxidation-related genes, such as *Cpt1* and *Acox1* in AML12 cells (Fig. 6H). *Lpin1* overexpression increased the expression of PGC1α and PPARα in AML12 cells (Fig. 6I). The suppressive effect of recombinant mouse CXCL5 on PPARα and FA oxidation genes was reversed by *Lpin1* overexpression (Fig. 6J), indicating that LPIN1 is a critical mediator of the CXCL6-induced suppression of FA oxidation. These findings demonstrate that CXCL6 suppresses hepatic FA oxidation by inhibiting GR-mediated *Lpin1* transcription, thereby attenuating PPARα and FA oxidation gene expression.

### LPIN1 mediates the protective effect of *Cxcl5* deficiency against AMLN diet-induced MASH progression

**Figure 7:**
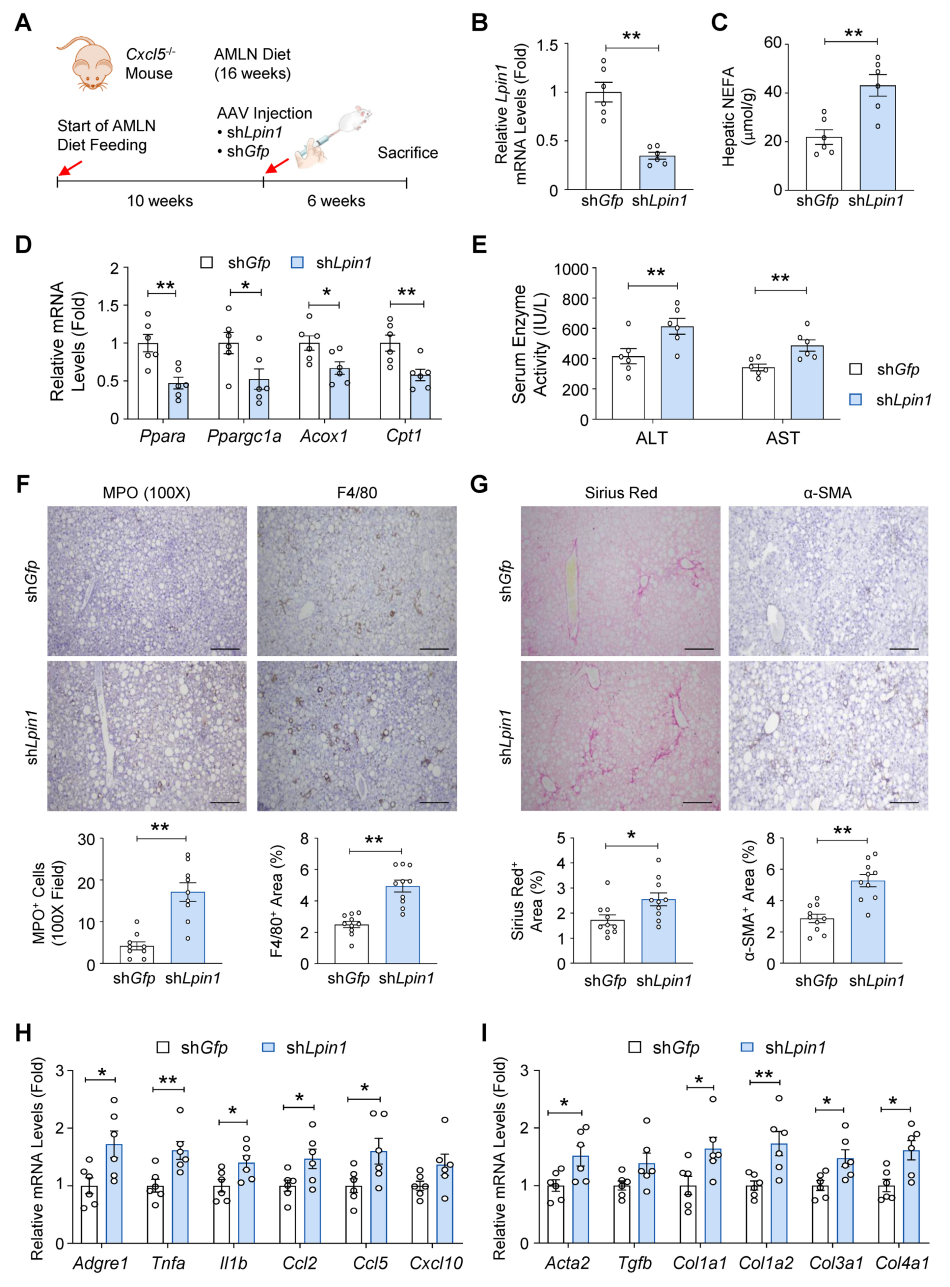
** Hepatic *Lpin1* knockdown reverses the protective effect of *Cxcl5* deficiency in AMLN diet-induced MASH.** (A) Male *Cxcl5*-deficient mice were fed an AMLN diet for 16 weeks. At week 10, mice were injected with AAV expressing shRNA (2 X 10^11^ genome copies per mouse) targeting *Lpin1* (sh*Lpin1*) or *Gfp* (sh*Gfp*). (B) Hepatic *Lpin1* mRNA levels were assessed by RT-qPCR. (C) Hepatic NEFA levels were quantified. (D) Hepatic mRNA levels of *Ppara, Ppargc1a, Acox1*, and *Cpt1* were assessed with RT-qPCR. (E) Serum ALT and AST levels. (F) Paraffin-embedded liver tissues were subjected to MPO staining and F4/80 staining (top). The number of MPO-positive cells per 100X field and the area positive for F4/80 staining were quantified (bottom) (G) Paraffin-embedded liver tissues were subjected to Sirius Red staining and α-SMA staining (top). The areas positive for Sirius Red staining and α-SMA staining were quantified (bottom). Scale bars indicate 200 μm. (H, I) Liver tissues were subjected to RT-qPCR analysis of proinflammatory genes (panel H) and fibrogenic genes (panel I). Values represent mean ± SEM. Statistical significance was assessed using Student's *t*-tests (*p < 0.05, **p < 0.01).

To investigate the *in vivo* relevance of LPIN1 in MASH pathogenesis, we examined whether *Lpin1* knockdown counteracts the protective effects of *Cxcl5* deletion. *Cxcl5*-deficient mice were fed an AMLN diet for 16 weeks. At week 10, they were injected with an AAV expressing shRNA targeting *Lpin1* (sh*Lpin1*) to knockdown *Lpin1* or a control shRNA against *Gfp* (sh*Gfp*) (Fig. 7A). RT-qPCR and immunoblot analyses confirmed the efficient knockdown of *Lpin1* in the liver (Fig. 7B and Fig. S13A). *Lpin1* knockdown increased hepatic NEFA levels in *Cxcl5*-deficient mice (Fig. 7C), whereas liver weight and triglyceride levels were not significantly altered (Fig. S13B). *Lpin1* knockdown suppressed *Ppara* and its downstream genes involved in FA oxidation (Fig. 7D). Serum ALT and AST levels were increased by *Lpin1* knockdown (Fig. 7E), indicating that *Lpin1* knockdown exacerbated liver injury in *Cxcl5*-deficient mice. Additionally, immunohistochemical analysis of MPO and F4/80 showed that hepatic recruitment of neutrophils and macrophages was enhanced in *Cxcl5-*deficient mice following *Lpin1* knockdown (Fig. 7F). Hepatic fibrosis was exacerbated by *Lpin1* knockdown in *Cxcl5*-deficient mice as shown by Sirius Red staining and immunohistochemical analysis of α-SMA (Fig. 7G). Consistent with these histological findings, the expression of inflammatory and fibrogenic genes was elevated by the knockdown of *Lpin1* (Figs. 7H-7I). Collectively, shRNA-mediated *Lpin1* knockdown exacerbated MASH phenotypes in *Cxcl5*-deficient mice, indicating that LPIN1 upregulation plays a critical role in alleviating MASH caused by *Cxcl5* deficiency.

### CXCL6 expression negatively correlates with LPIN1 in human MASH

**Figure 8:**
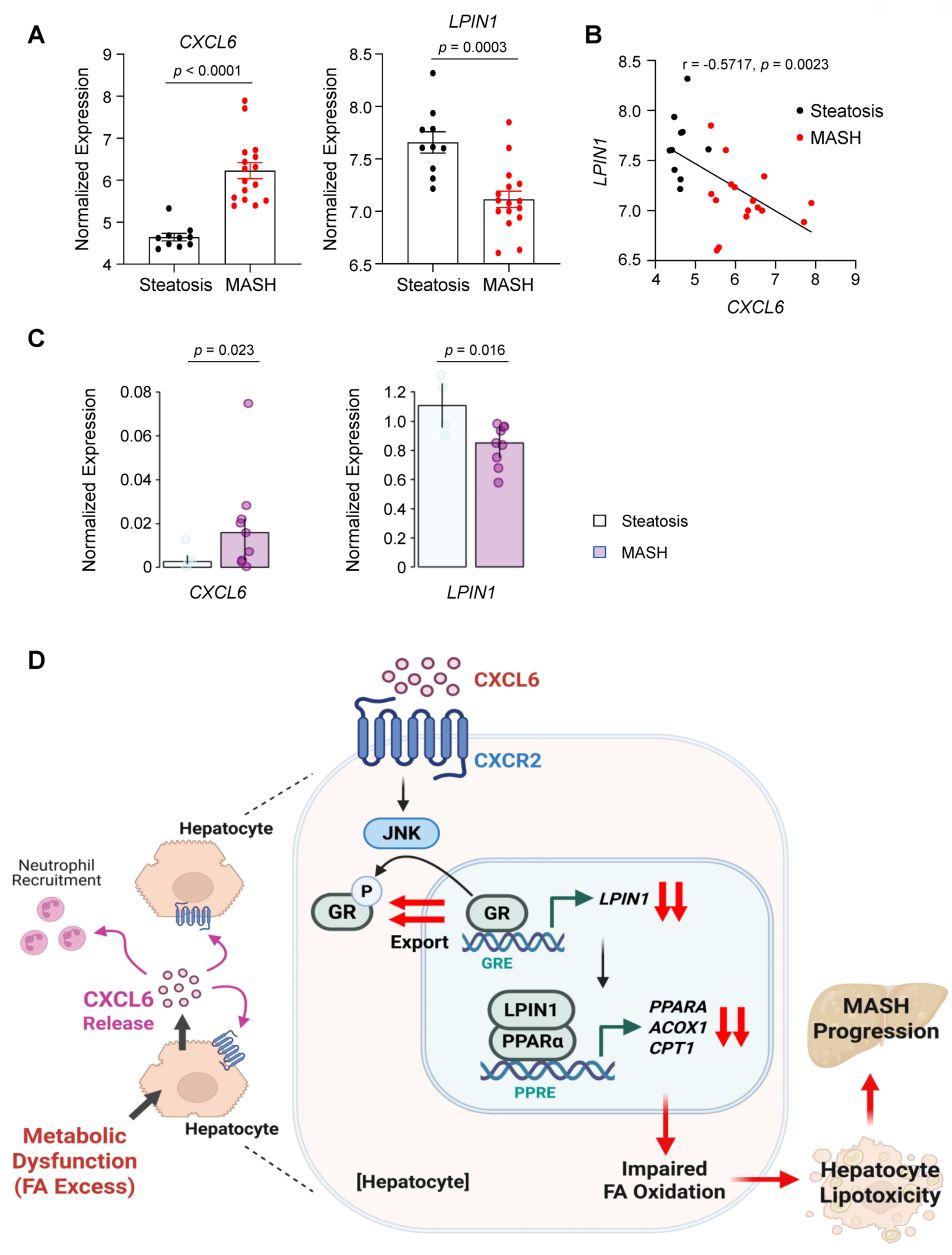
** CXCL6 expression is inversely correlated with LPIN1 in human MASH.** (A) Normalized expression levels of *CXCL6* and *LPIN1* in human liver samples from individuals with steatosis (*n* = 10) and MASH (*n* = 16) (dataset: E-MEXP-3291). Data are presented as mean ± SEM. Statistical significance was assessed using the Mann-Whitney U test. (B) Pearson correlation analysis showing an inverse relationship between *CXCL6* and *LPIN1* based on normalized log_2_ expression values. (C) Expression of *CXCL6* and *LPIN1* in hepatocytes from patients with steatosis (*n* = 4) and MASH (*n* = 9), analyzed using publicly available single-nucleus RNA-seq data (GSE244832). Pseudobulk profiles were generated by aggregating cell-level counts for each sample, followed by differential expression analysis using the Wald test. (D) Proposed model illustrating CXCL6-mediated suppression of LPIN1 and PPARα, thereby promoting lipotoxicity and MASH progression.

To determine whether LPIN1 expression was downregulated during the progression from steatosis to MASH, we analyzed publicly available microarray data from human liver samples (E-MEXP-3291) 32. *CXCL6* expression was significantly elevated in MASH (*n* = 16) compared with steatosis (*n* = 10), whereas the expression of *LPIN1* was reduced in MASH (Fig. 8A). Furthermore, the expression of *LPIN1* was negatively correlated with *CXCL6* expression in the liver. (Fig. 8B). To further assess the hepatocyte-specific expression patterns, we examined publicly available single-nucleus RNA-seq data (GSE244832) from patients with steatosis (*n* = 4) and MASH (*n* = 9) (Figs. S14A-S14C). Consistently, CXCL6 expression was elevated, and LPIN1 expression was reduced in hepatocytes from MASH samples compared with those from steatosis samples (Fig. 8C). Collectively, these results indicate that the suppressive effect of CXCL6 on LPIN1 in hepatocytes observed in experimental models is also evident in human MASH pathogenesis.

## Discussion

In the liver, the neutrophil chemoattractant CXCL6 is primarily produced by hepatocytes and is significantly upregulated during MASH pathogenesis. While CXCL6 is known to mediate neutrophil infiltration by interacting with the CXCR2 receptor on neutrophils, the direct impact of CXCL6 on hepatocytes, which also express CXCR2 33, 34, has been underexplored in the context of MASH development.

Hepatic steatosis is characterized by the excessive accumulation of FA in hepatocytes. Our data demonstrate that FA overload induces CXCL6 expression in hepatocytes. Importantly, this upregulation establishes a pathogenic positive feedback loop within hepatocytes: CXCL6 suppresses FA oxidation and aggravates lipotoxicity, thereby accelerating the progression from steatosis to MASH. Consistent with this, hepatic overexpression of *Cxcl5* (the murine homolog of *CXCL6*) exacerbated lipotoxicity and was sufficient to drive the progression to MASH in mice. Conversely, *Cxcl5* deletion ameliorated diet-induced MASH development.

Mechanistic analyses further elucidated the pivotal role of CXCL6 in MASH pathogenesis (Fig. 8D). We identified LPIN1 as a key mediator linking CXCL6 to PPARα inhibition and lipotoxicity. Previous studies established that LPIN1 activates PPARα by promoting its transcription and acting as a transcriptional coactivator 4. Extending this knowledge, we demonstrated that CXCL6 signaling inhibits LPIN1, which subsequently suppresses the expression of PPARα and downstream FA oxidation genes. This was confirmed through gain- and loss-of-function experiments targeting LPIN1 both *in vitro* and *in vivo*. Pharmacological inhibition revealed that CXCL6 suppresses LPIN1 and PPARα via CXCR2 activation. Furthermore, we highlighted the phosphorylated GR as a key downstream mediator of CXCR2 that contributes to the suppression of LPIN1 and PPARα.

Previous studies have identified functional GREs in both mouse *Lpin1* and human *LPIN1* promoters, suggesting that GR has the potential to induce LPIN1 and FA oxidation 20, 35. However, the precise role of GR in LPIN1-mediated FA oxidation and MASH pathogenesis remains poorly understood. Moreover, it is unclear how chemokines modulate GR activity in this context. Here, we demonstrate that CXCL6 promotes the inhibitory phosphorylation of GR at Ser226. The transcriptional activity of GR is regulated by phosphorylation at multiple sites, including Ser134, Ser211, and Ser226 28. Among these, Ser226 phosphorylation, mediated by JNK 26, 27, 29, reduces GR transcriptional activity and promotes the nuclear export of GR. Although CXCR2 activation by CXCL6 also triggers p38 activation 31, the role of p38 in Ser226 phosphorylation remains controversial 25, 29. Therefore, further studies utilizing selective inhibitors are required to clarify the relative contributions of JNK and p38 to CXCL6-induced GR inhibition.

Although the current study emphasizes the role of CXCL6 in dysregulating FA metabolism and aggravating hepatocyte lipotoxicity, the contribution of CXCL6-recruited neutrophils to MASH development cannot be entirely excluded. Our co-culture experiments demonstrated that neutrophils recruited by CXCL6 failed to directly impair FA oxidation in hepatocytes. Nevertheless, neutrophils and other immune cells may release cytokines (e.g., TNF-α, IL-1β, IL-17), which activate stress kinases, promote hepatocyte damage, and amplify inflammation. In addition, ROS released by neutrophils and macrophages exacerbates hepatocyte injury and promotes inflammation. Future studies should determine the extent to which neutrophils are involved in MASH pathogenesis, potentially by examining whether neutrophil depletion mitigates MASH induced by *Cxcl5* overexpression.

We observed that LPIN1 suppression by *Cxcl5* overexpression or AAV-sh*Lpin1* infection increases hepatic NEFA levels without elevating triglyceride levels. Beyond its role as a PPARα coactivator, LPIN1 functions as PAP that catalyzes the conversion of phosphatidic acid to diacylglycerol 3. Therefore, reduced PAP activity in *Cxcl5*-deficient mice may impair the conversion of FAs into neutral, nontoxic triglycerides. This impairment, combined with suppressed FA oxidation, likely promotes the accumulation of toxic FAs in the liver. Consistently, *Cxcl5* deletion lowered hepatic NEFA levels, while leaving hepatic triglyceride levels largely unchanged. Collectively, these findings suggest that LPIN1 induction may represent a promising therapeutic strategy to mitigate MASH pathogenesis by simultaneously enhancing FA oxidation and channeling FAs toward safe storage as triglycerides.

Single cell RNA-seq data from human livers demonstrate that hepatocytes are the major producer of CXCL6 in both healthy and cirrhotic tissues. In our pure hepatocyte cultures, *Cxcl5* knockout reduced *Lpin1* expression, while recombinant mouse CXCL5 treatment elevated it. Moreover, single nucleus RNA-seq analysis (GSE244832) revealed that CXCL6 was upregulated, while LPIN1 was downregulated in hepatocytes from MASH samples compared with steatosis samples. These findings suggest that CXCL6, primarily released by hepatocytes, inhibits the expression of LPIN1 in a cell autonomous fashion, presumably by interacting with CXCR2 on hepatocytes. This hypothesis is further supported by the autocrine functions of other chemokines, such as CCL2, which also contributes to lipid dysregulation via PPARα inhibition and dysregulation of fatty acid homeostasis in hepatocytes 36.

Our study demonstrates that CXCL6 suppresses LPIN1 and PPARα via CXCR2. Because CXCR2 interacts with multiple neutrophil-recruiting chemokines (e.g., CXCL1, CXCL2, CXCL8), and because we observed that CXCL1 treatment inhibited LPIN1 and PPARα in a manner comparable to CXCL6, a question arises regarding the relative contribution of CXCL6. Circulating CXCL5 levels are markedly higher than CXCL1 levels in mice under MASH-prone conditions 37 (Fig. 2B and Fig. S4A), underscoring the predominance of CXCL5 in this model. Accordingly, *Cxcl5* deletion alone markedly increased hepatic LPIN1 and PPARα expression in mice subjected to a MASH-inducing diet. Furthermore, PA stimulation elicited greater induction of *Cxcl5* in AML12 cells compared with other chemokines such as *Cxcl1* and *Cxcl2*. Collectively, these findings indicate that lipotoxic upregulation of *Cxcl5* plays a dominant role in disrupting FA metabolism and aggravating lipotoxicity in mice. However, in clinical settings, the precise contribution of human CXCL6 relative to other CXCR2 ligands remains uncertain, underscoring an important direction for future investigation.

Several studies have investigated the potential role of CXCL6 in MASH pathogenesis, although certain aspects remain to be further clarified. Zou et al. reported that siRNA-mediated knockdown of *CXCL6* reduced lipid accumulation and cell death in oleic acid-treated L02 cells 38. However, L02 cells have been reported to contain HeLa-derived components rather than representing authentic hepatocytes 39, which may limit the physiological relevance of these findings. Moreover, the study did not assess the role of CXCL6 in MASH development *in vivo*. Qi et al. demonstrated that a neutralizing antibody against mouse CXCL5 attenuated methionine choline-deficient (MCD) diet-induced liver injury in mice 40. In that study, CXCL5 was proposed to indirectly promote hepatocyte injury through Kupffer cell activation, which differs from our observation that CXCL6 directly induces hepatocyte lipotoxicity. In addition, MCD diet model does not fully recapitulate the metabolic dysfunction characteristic of human MASH, and the authors did not validate CXCL5 function using genetic mouse models. Building on these prior observations, the current study provides *in vivo* evidence from *Cxcl5* knockout mice and adenovirus-driven *Cxcl5* overexpression that CXCL6 promotes hepatocyte lipotoxicity and drives MASH development under metabolically relevant dietary conditions. Mechanistically, we further identify that CXCL6 exacerbates lipotoxicity via the GR-LPIN1-PPARα axis.

Finally, our findings underscore the clinical relevance of CXCL6 in MASH pathogenesis. Analyses of public datasets reveal a distinct expression profile in patients with MASH compared with simple steatosis: *CXCL6* is markedly upregulated, whereas *LPIN1* is consistently downregulated. This inverse relationship, evidenced by a significant negative correlation in human liver tissues, supports the translational relevance of our mechanistic finding that CXCL6 suppresses the LPIN1-PPARα pathway. Collectively, these data suggest that CXCL6-mediated inhibition of the LPIN1-PPARα axis represents a clinically relevant mechanism in MASH progression. Consequently, therapeutic interventions that attenuate CXCL6 signaling to restore LPIN1 and PPARα activity may offer a promising strategy for treating MASH.

## Supplementary Material

Supplementary figures and table.
