# Supplementary material for: CXCL6 exacerbates metabolic dysfunction-associated steatohepatitis by suppressing LPIN1-mediated fatty acid oxidation in hepatocytes
Source: Int J Biol Sci. 2026 May 1;22(10):5015–35. doi: 10.7150/ijbs.129358 (PMC13215063; doi:10.7150/ijbs.129358)
Supplement: Supplementary file 1 — Supplementary figures and table. [file ijbsv22p5015s1.pdf]

Supplementary Information

CXCL6 exacerbates metabolic dysfunction-associated steatohepatitis by suppressing LPIN1-mediated fatty acid oxidation in hepatocytes.

Ye Eun Cho,[1,#] Min-Ju Kim,[1,#] Yeonsoo Kim,[1] Hyeokjin Lim,[1] Yunseo Park,[1] Sangok Kim,[2] Seung-Jin Kim,[3] Jin-Wook Yoo,[1] Seungjin Ryu,[4] Parkyong Song,[5] Changwan Hong,[6,7] Yong He,[8] Haeseung Lee,[1] Je-Yoel Cho,[9,10] Seonghwan Hwang[1,*]

[1]College of Pharmacy and Research Institute for Drug Development, Pusan National University, Busan 46241, Republic of Korea

[2]Korea Bioinformation Center, Korea Research Institute of Bioscience & Biotechnology, Daejeon 34141, Republic of Korea

[3]Department of Biochemistry, College of Natural Sciences, Kangwon National University, Chuncheon 24341, Republic of Korea

[4]Department of Biochemistry, Chung-Ang University College of Medicine, Seoul 06974, Republic of Korea

[5]Department of Convergence Medicine, Pusan National University School of Medicine, Yangsan 50612, Republic of Korea

[6]Department of Anatomy, Pusan National University School of Medicine, Yangsan 50612, Republic of Korea

[7]Department of Convergence Medical Science, Pusan National University School of Medicine, Yangsan 50612, Republic of Korea

[8]State Key Laboratory of Drug Research, Shanghai Institute of Materia Medica (SIMM), Chinese Academy of Sciences, Shanghai 201203, China

[9]Department of Biochemistry, Brain Korea 21 Project and Research Institute for Veterinary Science, Seoul National University, College of Veterinary Medicine, Seoul 08826, Republic of Korea.

[10]Comparative Medicine Disease Research Center (CDRC), Science Research Center (SRC), Seoul National University, Seoul 08826, Republic of Korea

[#]YEC and MJK contributed equally to this work.

***Corresponding author**: Seonghwan Hwang, Ph.D.

E-mail: shhwang@pusan.ac.kr; Tel.: +82-51-510-2817; Fax: +82-51-513-6754.

Supplementary Table S1. Primer sequences for real time quantitative PCR

Gene Name	Forward (5'-3')	Reverse (5'-3')
Apob	CGTGGGCTCCAGCATTCTA	TCACCAGTCATTTCTGCCTTTG
Gapdh	AGCAGCCGCATCTTCTTGTGCAGTG	GGCCTTGACTGTGCCGTTGAATTT
Cxcl1	ACTGCACCCAAACCGAAGTC	TGGGGACACCTTTTAGCATCTT
Cxcl2	CATCCAGAGCTTGAGTGTGACG	GGCTTCAGGGTCAAGGCAAACT
Cxcl5	CCGCTGGCATTTCTGTTGCTGT	CAGGGATCACCTCCAAATTAGCG
Ccl2	TCTGGACCCATTCCTTCTTGG	TCAGCCAGATGCAGTTAACGC
Tnfa	AGGCTGCCCCGACTACGT	GACTTTCTCCTGGTATGAGATAGCAAA
Il1b	TCGCTCAGGGTCACAAGAAA	CATCAGAGGCAAGGAGGAAAAC
Adgre1	CTTTGGCTATGGGCTTCCAGTC	GCAAGGAGGACAGAGTTTATCGTG
Tgfb	CAACCCAGGTCCTTCCTAAA	GGAGAGCCCTGGATACCAAC
Acta2	TCCTGACGCTGAAGTATCCGATA	GGTGCCAGATCTTTTCCATGTC
Col1a1	TAGGCCATTGTGTATGCAGC	ACATGTTCAGCTTTGTGGACC
Col1a2	GGTGAGCCTGGTCAAACGG	ACTGTGTCCTTTCACGCCTTT
Col3a1	TAGGACTGACCAAGGTGGCT	GGAACCTGGTTTCTTCTCACC
Col4a1	CACATTTTCCACAGCCAGAG	GTCTGGCTTCTGCTGCTCTT
Ncf1	TCCTCTTCAACAGCAGCGTA	CTATCTGGAGCCCCTTGACA
Ncf2	TCTATCAGCTGGTTCCCACG	TGGCCTACTTCCAGAGAGGA
Ncf4	ATCGTCTGGAAGCTGCTCAA	CCCATCCATCTGCTTTTCTG
Cyba	ATGGAGCGATGTGGACAGAAG	TAGATCACACTGGCAATGGCC
Cybb	GACCATTGCAAGTGAACACCC	AAATGAAGTGGACTCCACGCG
Lpin1	CTCCGCTCCCGAGAGAAAG	TCATGTGCAAATCCACGGACT
Ppargc1a	TATGGAGTGACATAGAGTGTGCT	CCACTTCAATCCACCCAGAAAG
Acox1	TAACTTCCTCACTCGAAGCCA	AGTTCCATGACCCATCTCTGTC
Cpt1	AGATCAATCGGACCCTAGACAC	CAGCGAGTAGCGCATAGTCA
Ppara	AACATCGAGTGTCGAATATGTGG	CCGAATAGTTCGCCGAAAGAA
Mt-Co1	CCAGTGCTAGCCGCAGGCAT	GCTGGTAGAGAATTGGGTCCCCTCC
Ucp2	TAAAGGTCCGCTTCCAGGCTCA	ACGGGCAACATTGGGAGAAGTC
Cycs	GAGGCAAGCATAAGACTGGACC	ACTCCATCAGGGTATCCTCTCC
Atp5f1b	CTCTGACTGGTTTGACCGTTGC	TGGTAGCCTACAGCAGAAGGGA
Tfam	GAGGCAAAGGATGATTCGGCTC	CGAATCCTATCATCTTTAGCAAGC
GAPDH	GCCCCAGCGTCAAAGGT	GGCATCCTGGGCTACACTGA
CXCL1	GAAAGCTTGCCTCAATCCTG	CTTCCTCCTCCCTTCTGGTC
CXCL2	GGCAGAAAGCTTGTCTCAACCC	CTCCTTCAGGAACAGCCACCAA
CXCL6	GGGAAGCAAGTTTGTCTGGACC	AAACTGCTCCGCTGAAGACTGG
CXCL8	GAGAGTGATTGAGAGTGGACCAC	CACAACCCTCTGCACCCAGTTT

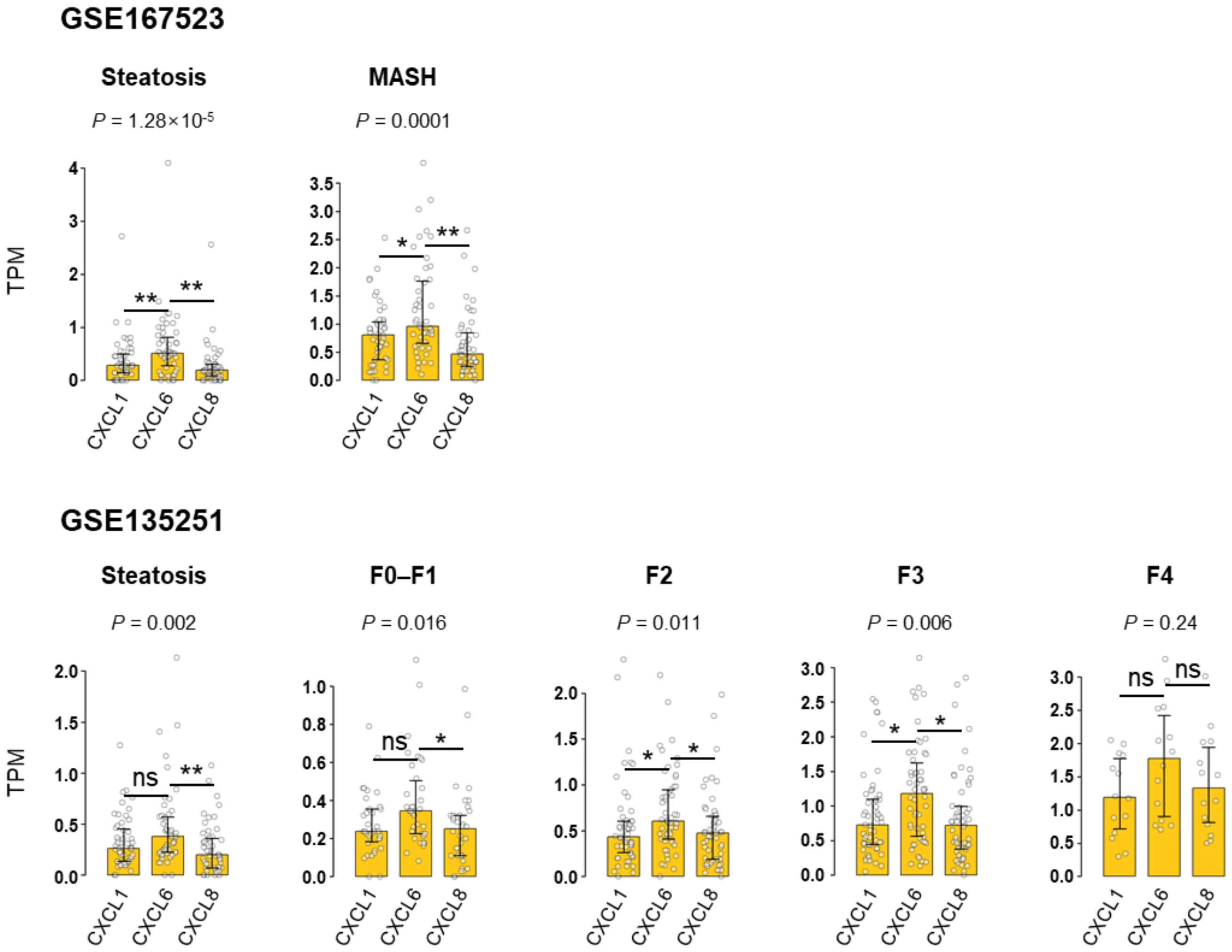

Figure S1. Predominant expression of CXCL6 among neutrophil-recruiting CXCR2 ligand chemokines in human livers. The expression levels of *CXCL1*, *CXCL6*, and *CXCL8* were compared across different disease stages in two independent human liver transcriptomic datasets (GSE167523 and GSE135251). GSE167523 includes steatosis and MASH samples, while GSE135251 includes steatosis and progressive fibrosis stages (F0–F1, F2, F3, and F4). Bars indicate median TPM values, and error bars represent the interquartile range (Q1–Q3). Statistical significance was assessed using the Kruskal-Wallis test followed by post-hoc pairwise comparisons (ns: not significant, *$p < 0.05$, **$p < 0.01$).

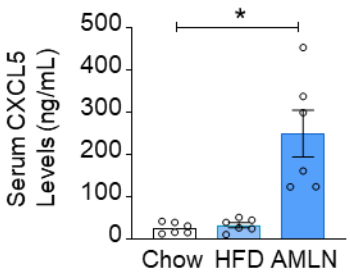

Figure S2. Elevation of serum CXCL5 levels in mice fed an AMLN diet. Male C57BL/6J mice (n = 6) were fed a chow diet (12 weeks), a high-fat diet (HFD, 12 weeks), or a gubra amylin (AMLN) diet (16 weeks). Serum CXCL5 levels were quantified by ELISA. Values represent mean ± SEM. Statistical significance was assessed using one-way analysis of variance (ANOVA), followed by Tukey's post-hoc test (*$p < 0.05$).

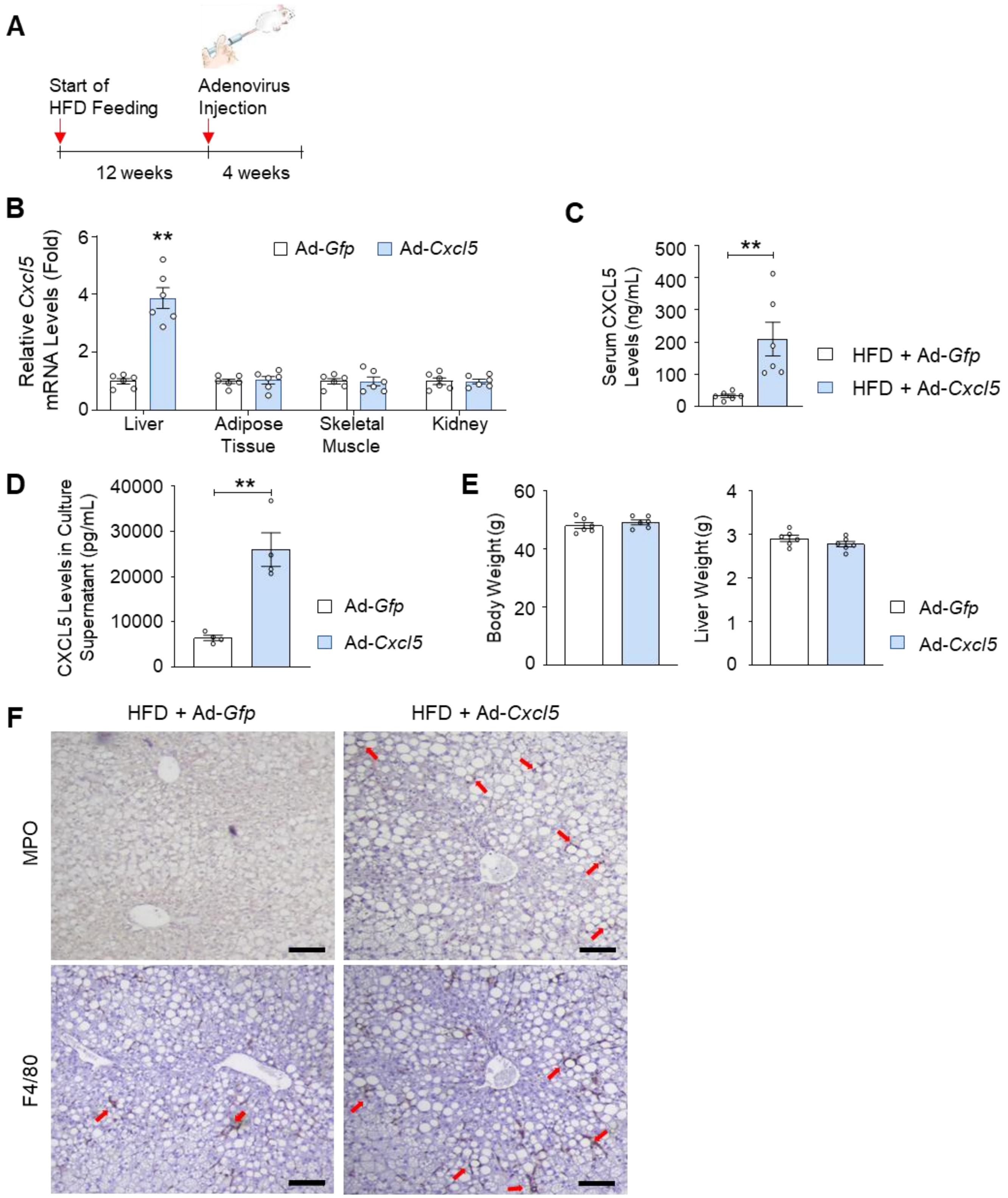

Figure S3. Characterization of *Cxcl5*-overexpressing mice fed a high-fat diet. (A) Male C57BL/6J mice (n = 6) were fed a high-fat diet for 16 weeks. At week 12, mice were injected with an adenovirus expressing *Cxcl5* (Ad-*Cxcl5*) or *Gfp* (Ad-*Gfp*) as a control. At week 16, mice were sacrificed. (B) RNA was extracted from the liver, adipose tissue, gastrocnemius, and kidney. *Cxcl5* transcript levels were assessed by RT-qPCR analysis. (C) Serum CXCL5 levels were quantified by ELISA. (D) Primary mouse hepatocytes isolated from Ad-*Cxcl5*-infected and Ad-*Gfp*-infected mice were cultured for 24 h. CXCL5 levels in culture supernatant were analyzed by ELISA. (E) Body and liver weights were measured at sacrifice. (F) Paraffin-embedded liver tissues were subjected to immunohistochemical analysis of myeloperoxidase (MPO) and F4/80. Scale bars indicate 200 μm. Red arrows indicate the MPO-positive cells for MPO staining images (top) and hepatic crown-like structure with F4/80 expression for F4/80 staining images (bottom). Values represent mean ± SEM. Statistical significance was assessed using Student's t-tests (**p < 0.01).

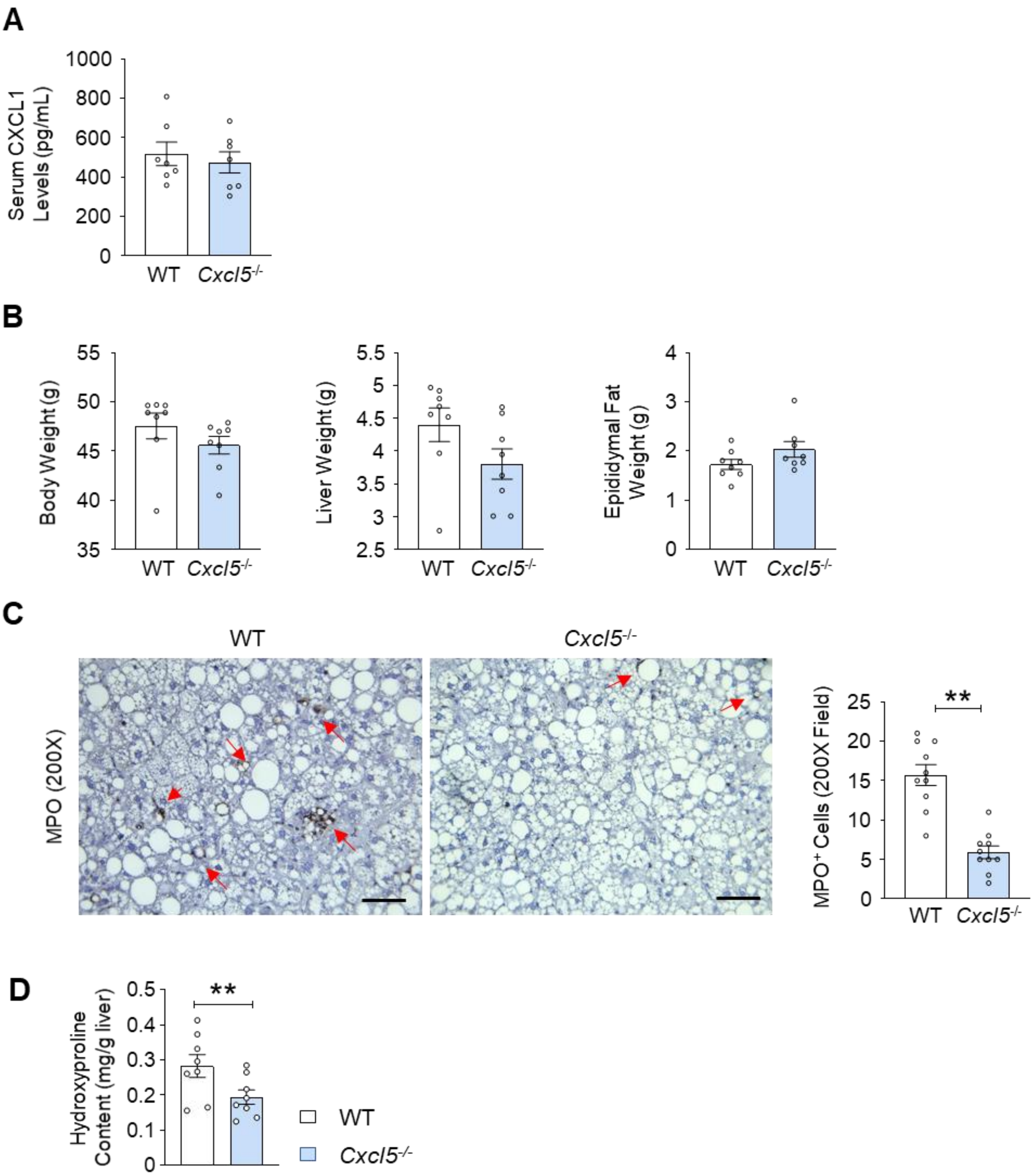

Figure S4. Characterization of *Cxcl5*-deficient mice fed an AMLN diet. *Cxcl5*-deficient mice and wild-type (WT) littermate controls were fed an AMLN diet for 20 weeks to induce MASH (n = 6). (A) Serum CXCL1 levels were measured by ELISA at sacrifice. (B) Body, liver, and epididymal fat weights were measured at sacrifice. (C) Paraffin-embedded liver tissues were subjected to MPO staining (left). The number of MPO-positive cells per 200X field was counted (right). (D) Hepatic hydroxyproline contents were quantified. Scale bars indicate 100 μm. Values represent mean ± SEM. Statistical significance was assessed using Student's t-tests (**$p < 0.01$).

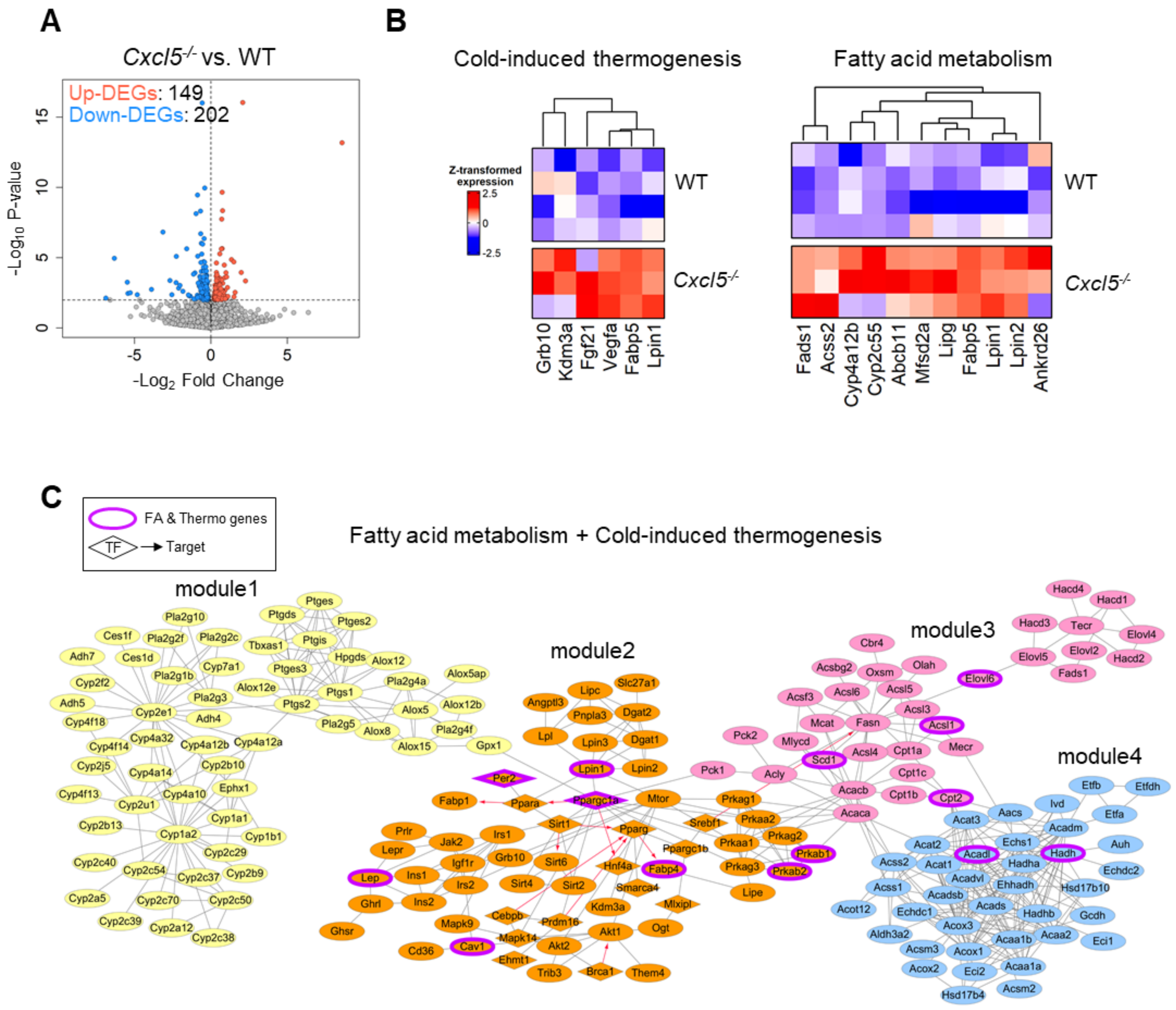

Figure S5. Transcriptomic alterations in *Cxcl5*-deficient mouse livers. (A) Volcano plot showing differentially expressed genes in the livers of *Cxcl5*$^{-/-}$ and wild-type (WT) mice. Up-regulated genes (n = 149) are shown in red, and down-regulated genes (n = 202) are shown in blue. The horizontal dashed line indicates the significance threshold (p < 0.05). (B) Heatmaps displaying z-score–normalized TPM values of genes involved in cold-induced thermogenesis (left) and fatty acid metabolism (right) pathways in WT and *Cxcl5*$^{-/-}$ mice. (C) Network analysis of genes involved in fatty acid metabolism and cold-induced thermogenesis. Genes are clustered into 4 modules based on functional relationships. Module colors indicate different functional gene clusters.

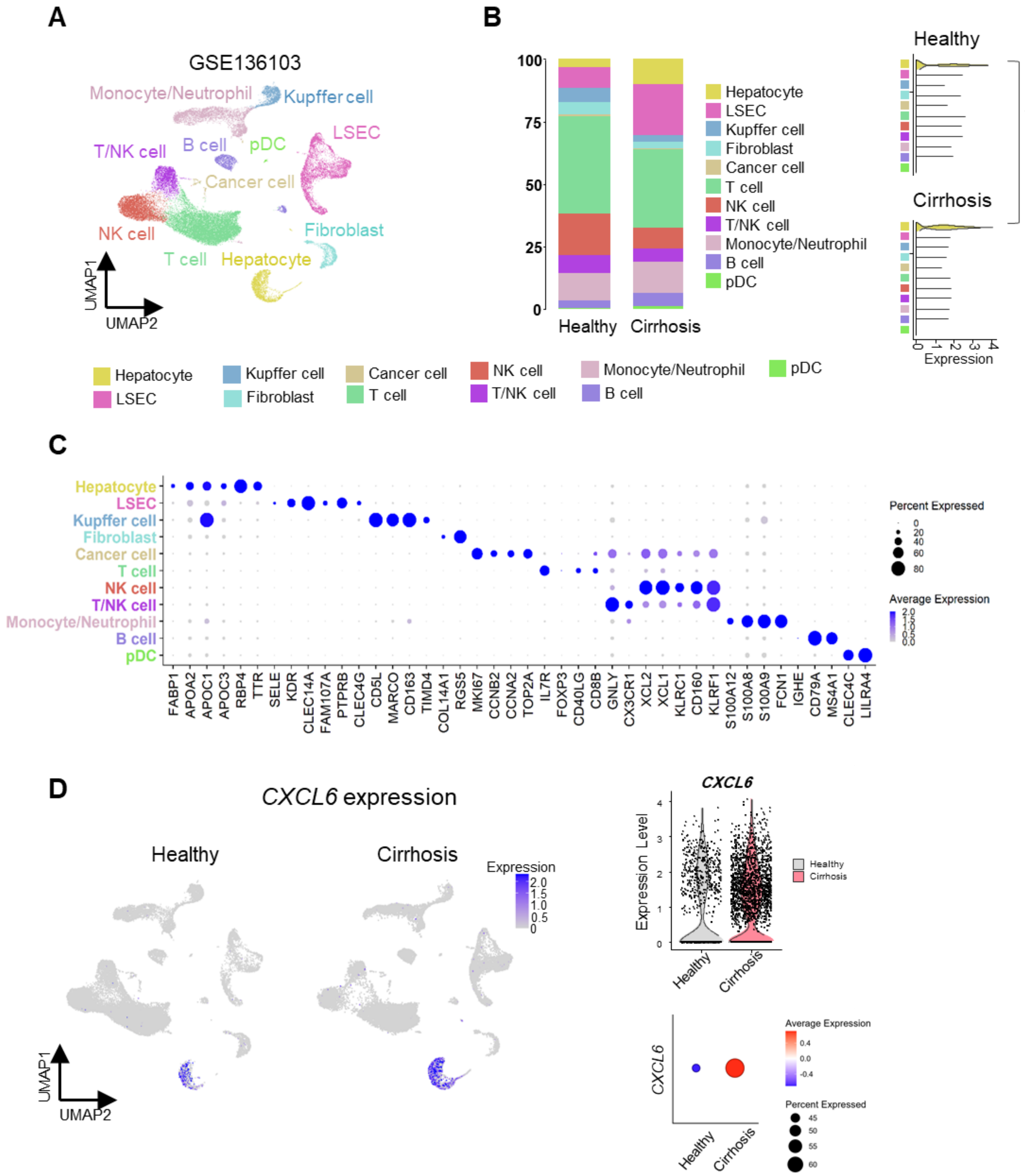

Figure S6. Hepatocyte-dominant CXCL6 expression and its elevation in cirrhotic livers. (A) UMAP visualization of single-cell RNA-seq data (GSE136103), showing distinct cell populations in human liver tissues. Cell types are color-coded and labeled. (B) Cell-type composition of healthy and cirrhotic liver samples. The proportion of each cell population is displayed as a stacked bar plot (left). The comparison of average gene expression across samples (right) shows a significant increase in hepatocyte-derived *CXCL6* expression in cirrhosis ($p < 0.001$). (C) Dot plot showing the expression of representative marker genes for each cell type. The size of the dots represents the percentage of cells expressing each gene, and the color intensity indicates the average expression level. (D) Expression pattern of *CXCL6* across liver cell types. UMAP plots display *CXCL6* expression in healthy and cirrhotic livers (left). Violin and dot plots (right) show increased *CXCL6* expression in hepatocytes from cirrhotic livers compared with healthy controls.

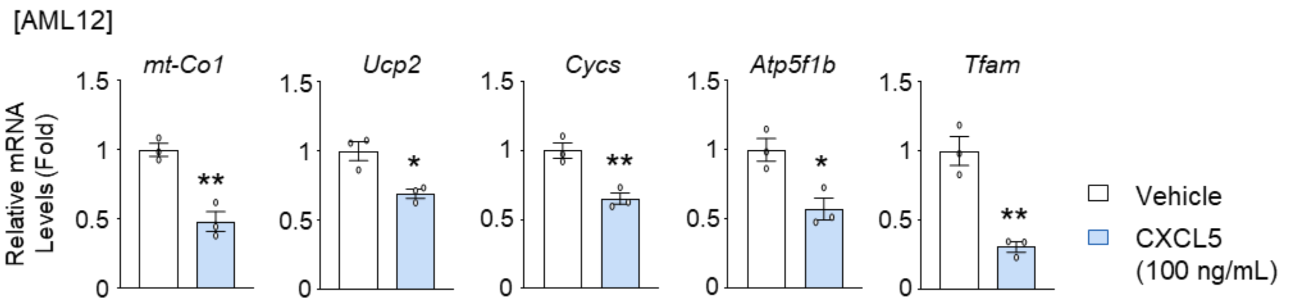

Figure S7. Effect of recombinant CXCL5 treatment on the expression of mitochondrial genes. AML12 cells were treated with vehicle or recombinant mouse CXCL5 for 24 h. mRNA levels of mitochondrial genes were quantified by RT-qPCR. Values represent mean ± SEM. Statistical significance was assessed using Student's *t*-tests (**p* < 0.05, ***p* < 0.01).

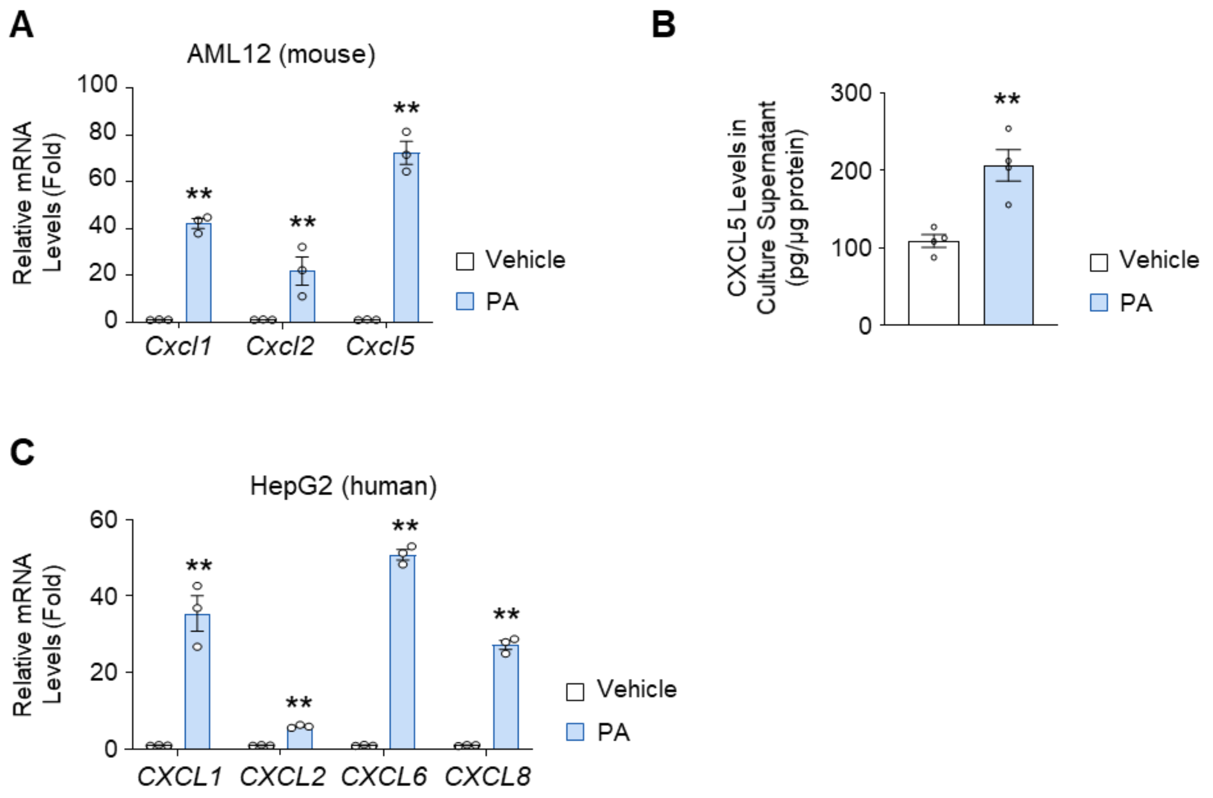

Figure S8. Induction of neutrophil-recruiting chemokines by palmitic acid (PA) in hepatocytes. (A) AML12 cells were treated with PA (0.4 mM) for 12 h. Transcript levels of *Cxcl1, Cxcl2,* and *Cxcl5* were assessed by RT-qPCR. (B) AML12 cells were treated with PA (0.4 mM) for 24 h. CXCL5 levels in the culture supernatant were measured by ELISA. (C) HepG2 cells were treated with PA (0.4 mM) for 6 h. Transcript levels of *CXCL1*, *CXCL2*, *CXCL6*, and *CXCL8* were assessed by RT-qPCR.

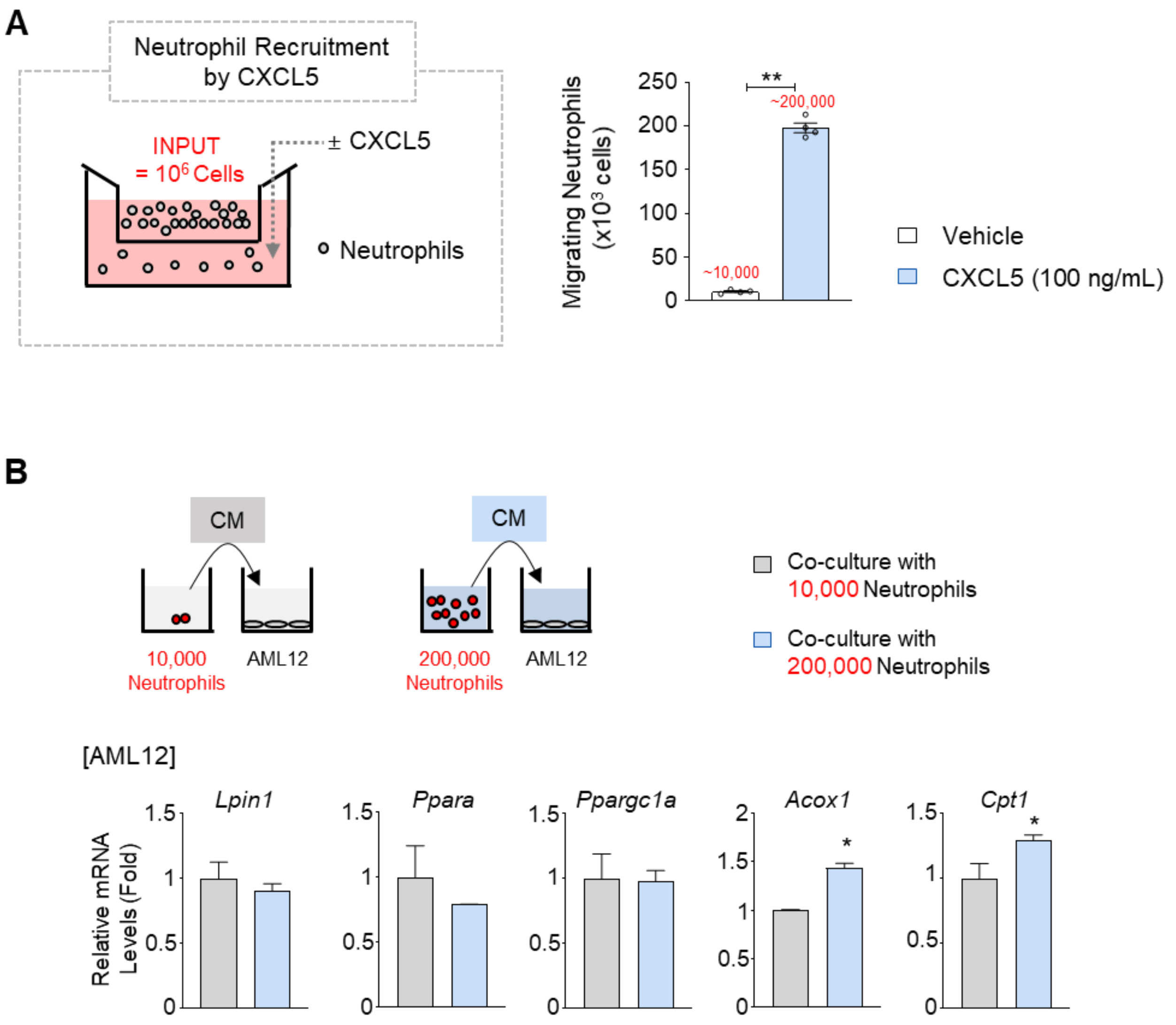

Figure S9. Effect of recruited neutrophils on the expression of LPIN1 and PPARα in AML12 hepatocytes. (A) Neutrophils (1 x 10^6 cells) were placed onto the insert (3 µm pore size) of a transwell system, and recombinant mouse CXCL5 (100 ng/mL) was added to the lower chamber, where AML12 cells were cultured. The number of neutrophils that migrated to the lower chamber (6 h) was quantified (n = 3). (B) Conditioned media (CM) were collected from neutrophils (10,000 or 200,000 cells) cultured for 6 h. AML12 cells were then cultured with the collected CM for 24 h, followed by RT-qPCR analysis of *Lpin1*, *Ppara*, *Ppargc1a*, *Acox1*, and *Cpt1*. Values represent mean ± SEM. Statistical significance was assessed using Student's *t*-tests (*$p < 0.05$).

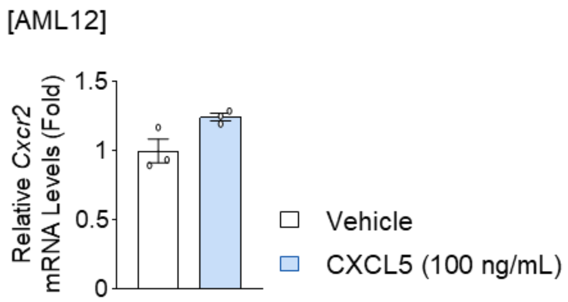

Figure S10. Effect of recombinant mouse CXCL5 on CXCR2 expression in AML12 cells. AML12 cells were retreated with recombinant mouse CXCL5 (100 ng/mL) for 24 h. *Cxcr2* mRNA levels were quantified by RT-qPCR. Values represent mean ± SEM. Statistical significance was assessed using Student's *t*-tests.

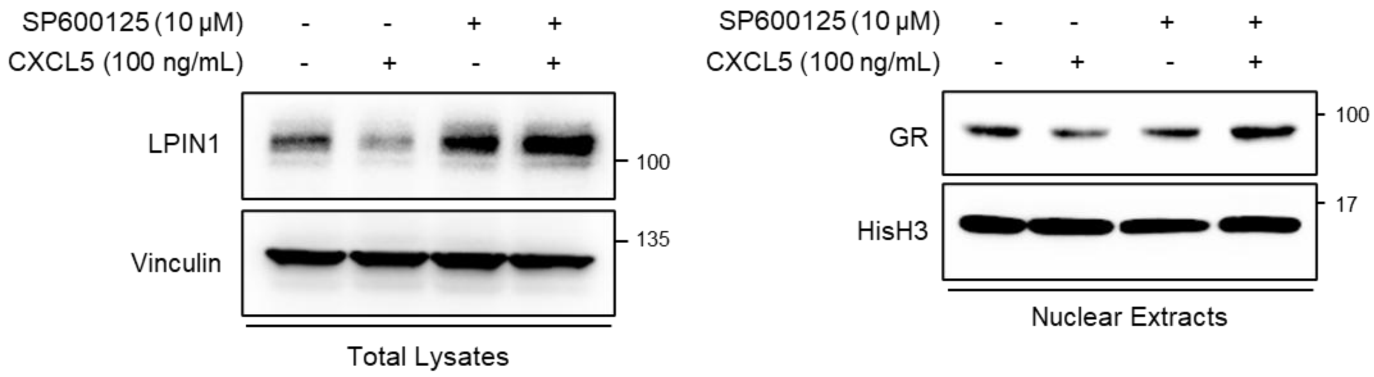

Figure S11. Attenuation of CXCL5-mediated suppression of LPIN1 and GR by pharmacological JNK inhibition. AML12 cells were pretreated with the JNK inhibitor SP600125 (10 μM) for 30 min and then treated with recombinant mouse CXCL5 (100 ng/mL) or vehicle for 24 h. Total lysates and nuclear extracts were subjected to immunoblot analysis.

A

[The promoter sequence of the mouse *Lpin1* gene]

LPIN1-421WT

```
TCCTTCCTGGCTCTGCTGACTGGTGGTGAATGTTAGCCAGGAGGGAGGCAGGA
TGTGGGAGTGCTAAGATTAGGAATTGATCCCTAGCTTGTGACAGGCACATCAG
GGGAAGAACTAATTGTAAACTGACATAAGGTCAAAACAGGGTCAGGCTGTGGA
ATTAGAAGACTTTGGCTGCCTACTCCCTGGAACGCCAAGGCAGACAGTCCAGT
CTGGGGAGGGATGGGATGGGAAGGGGGAGGCGGGGATATGAGAGGGAGGTGGG
AGGTGGGGTGGGGTGGGGGGGGGTCTGTGGGCTCTGAAAGCATCCCAGCAGC
ACGCTCCTCCAGAGTCCCCTGGCGTCGCCCCGGAGACTCCGCCTCCTTTCAGG
CCCCGCCCCTTGCCGTGCTTTAGCTGACAGCCGCTCCGGCCCGCTGTTTG
```

- Sequences highlighted in red indicate the glucocorticoid response element (GRE).

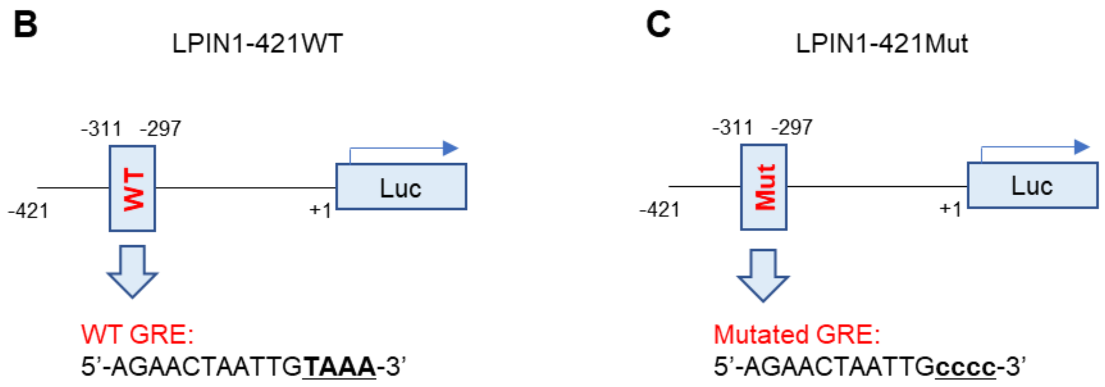

Figure S12. Analysis of the *Lpin1* promoter activity by luciferase reporter assays. (A) Nucleotide sequence of the proximal promoter region (-421 bp) of the mouse *Lpin1* gene. (B–C) Schematic diagrams of luciferase reporter constructs containing different *Lpin1* promoter regions. (B) Construct containing the wild-type (WT) glucocorticoid response element (GRE) within the *Lpin1* promoter. (C) Construct containing a mutated GRE, in which the last four nucleotides of the GRE sequence were replaced with cytosines.

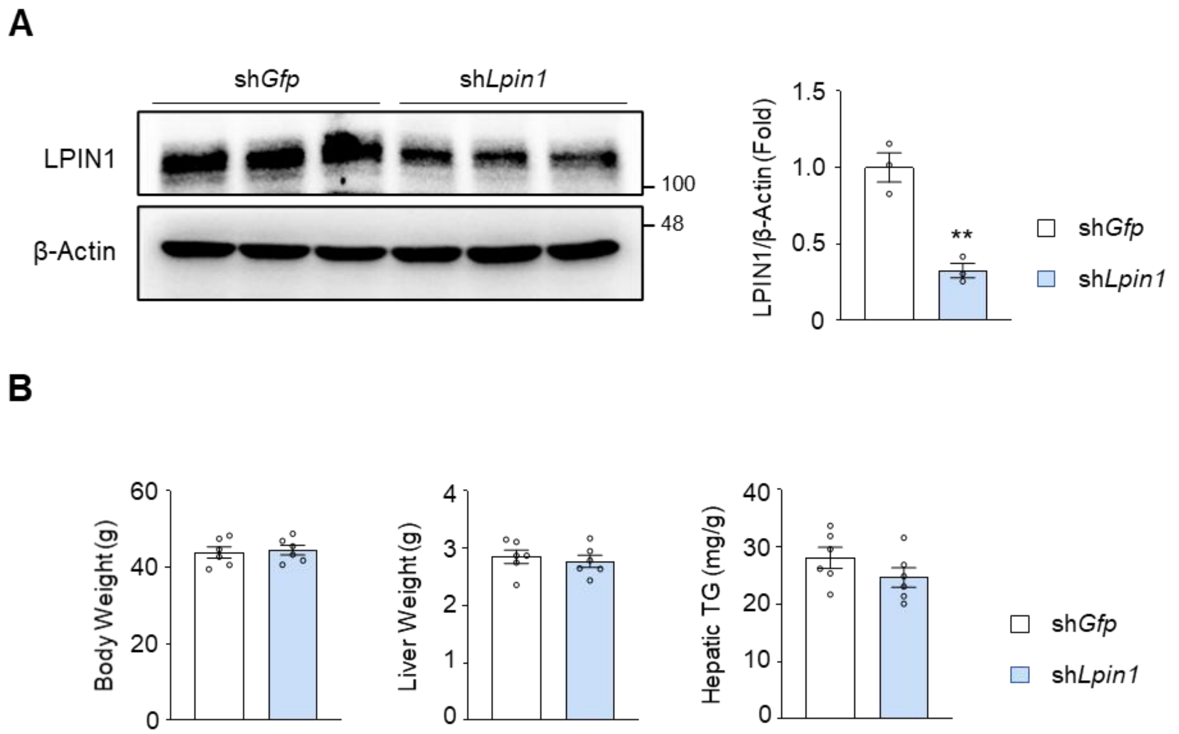

Figure S13. Characterization of *Cxcl5*-deficient mice with *Lpin1* knockdown. (A) Male *Cxcl5*-deficient mice were fed an AMLN diet for 16 weeks. At week 10, mice were injected with AAV expressing shRNA targeting *Lpin1* (sh*Lpin1*) or *Gfp* (sh*Gfp*). Total liver lysates were subjected to immunoblot analysis. (B) Body weight, liver weight, and hepatic triglyceride (TG) levels at sacrifice. Values represent mean ± SEM. Statistical significance was assessed using Student's *t*-tests.

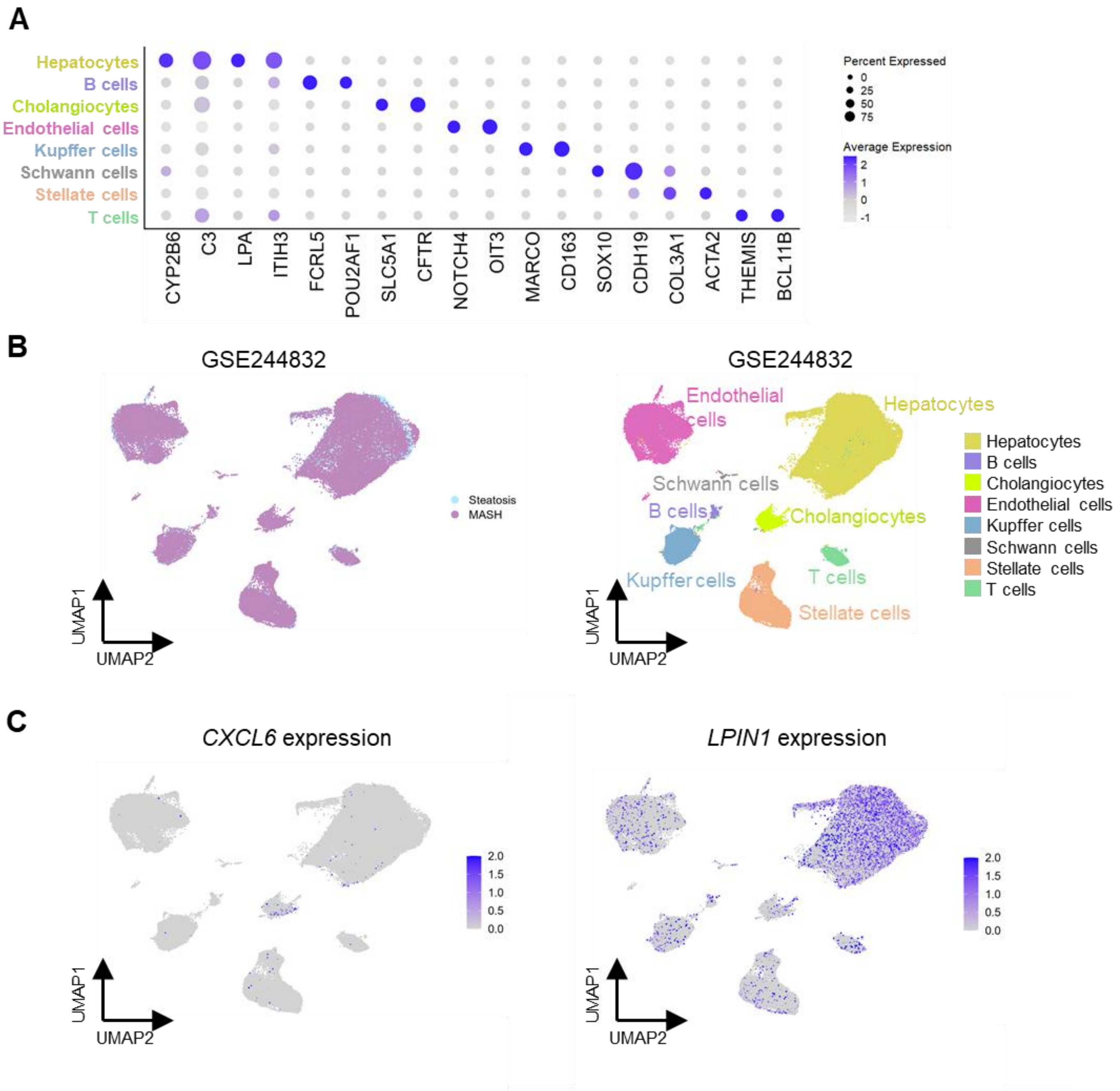

Figure S14. Opposite regulation of CXCL6 and LPIN1 in hepatocytes from patients with MASH. (A) Dot plot showing the expression of representative marker genes for each cell type in the single-nucleus RNA-seq dataset (GSE244832). The size of the dots represents the percentage of cells expressing each gene, and the color intensity indicates the average expression level. (B) UMAP visualization showing sample grouping by disease status (steatosis vs. MASH, left) and annotated cell identities (right). (C) Expression patterns of *CXCL6* and *LPIN1* across liver cell types.
